# Replicating dynamic humerus motion using an industrial robot

**DOI:** 10.1371/journal.pone.0242005

**Published:** 2020-11-09

**Authors:** Klevis Aliaj, Gentry M. Feeney, Balakumar Sundaralingam, Tucker Hermans, K. Bo Foreman, Kent N. Bachus, Heath B. Henninger

**Affiliations:** 1 Department of Orthopaedics, University of Utah, Salt Lake City, Utah, United States of America; 2 Department of Bimedical Engineering, University of Utah, Salt Lake City, Utah, United States of America; 3 School of Computing, University of Utah, Salt Lake City, Utah, United States of America; 4 Department of Mechanical Engineering, University of Utah, Salt Lake City, Utah, United States of America; 5 Department of Physical Therapy & Athletic Training, University of Utah, Salt Lake City, Utah, United States of America; 6 U.S. Department of Veterans Affairs, Salt Lake City, Utah, United States of America; National University of Ireland Galway, IRELAND

## Abstract

Transhumeral percutaneous osseointegrated prostheses provide upper-extremity amputees with increased range of motion, more natural movement patterns, and enhanced proprioception. However, direct skeletal attachment of the endoprosthesis elevates the risk of bone fracture, which could necessitate revision surgery or result in loss of the residual limb. Bone fracture loads are direction dependent, strain rate dependent, and load rate dependent. Furthermore, *in vivo*, bone experiences multiaxial loading. Yet, mechanical characterization of the bone-implant interface is still performed with simple uni- or bi-axial loading scenarios that do not replicate the dynamic multiaxial loading environment inherent in human motion. The objective of this investigation was to reproduce the dynamic multiaxial loading conditions that the humerus experiences *in vivo* by robotically replicating humeral kinematics of advanced activities of daily living typical of an active amputee population. Specifically, 115 jumping jack, 105 jogging, 15 jug lift, and 15 internal rotation trials—previously recorded via skin-marker motion capture—were replicated on an industrial robot and the resulting humeral trajectories were verified using an optical tracking system. To achieve this goal, a computational pipeline that accepts a motion capture trajectory as input and outputs a motion program for an industrial robot was implemented, validated, and made accessible via public code repositories. The industrial manipulator utilized in this study was able to robotically replicate over 95% of the aforementioned trials to within the characteristic error present in skin-marker derived motion capture datasets. This investigation demonstrates the ability to robotically replicate human motion that recapitulates the inertial forces and moments of high-speed, multiaxial activities for biomechanical and orthopaedic investigations. It also establishes a library of robotically replicated motions that can be utilized in future studies to characterize the interaction of prosthetic devices with the skeletal system, and introduces a computational pipeline for expanding this motion library.

## 1. Introduction

Universal Testing Machines (UTM) provide precise position/force control and measurement for biomechanical testing. Advanced UTMs can move at physiologically relevant speeds, but typically only in uni- or bi-axial configurations [1]. Alternatively, a robotic manipulator equipped with a universal force-moment sensor provides precise force, position, or hybrid control in all six spatial degrees-of-freedom (3 translations/rotations). Robotic manipulators have been utilized in joint simulators that enable investigations of the shoulder [2–4], hip [5, 6], knee [7–9], ankle [10, 11], spine [12], and elbow [13]. The proliferation of joint simulators has even warranted the creation of commercial software that enables their development from generic hardware [14].

**Fig 1:**
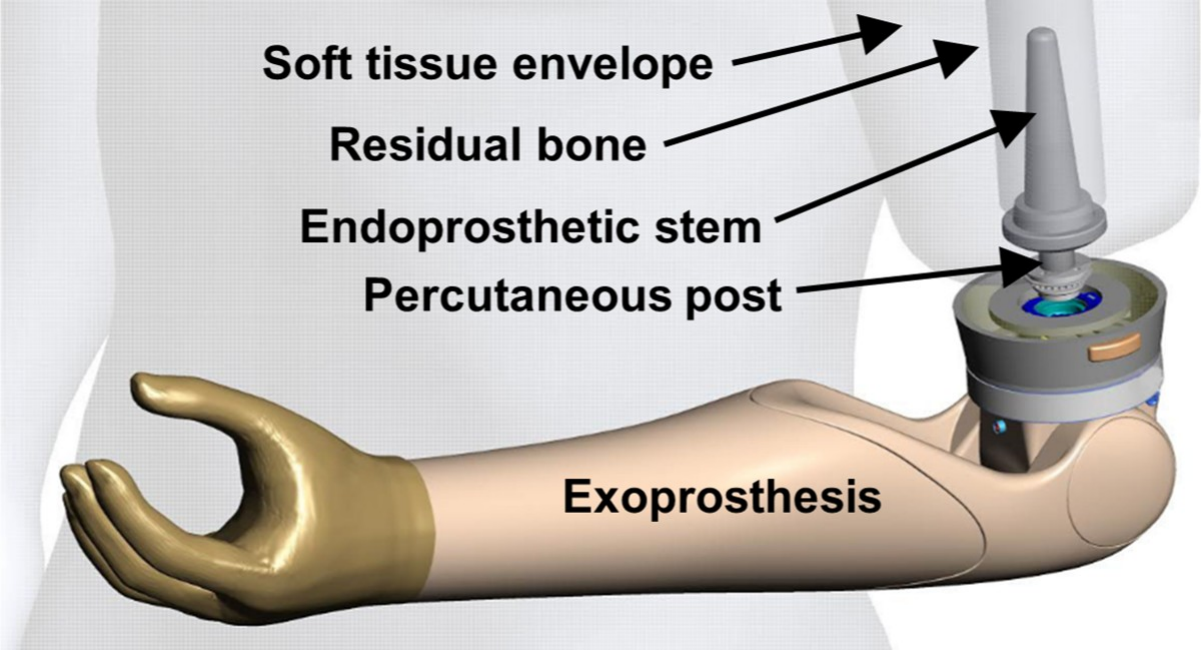
Schematic of a percutaneous osseointegrated prosthetic system for transhumeral amputation. An endoprosthesis placed in the residual humerus passes through the skin to support the exoprosthetic forearm and hand. This system obviates the need for typical socket suspension that interfaces to the soft tissues of the residual limb. Reprinted under a CC BY license, with permission from Motion Control Inc, a Fillauer Company.

In a typical experimental scenario, one segment of diarthrodial joint is rigidly fixed while the other is rotated and translated via the manipulator, although more complex designs exist [14, 15]. Most simulator studies are conducted quasi-statically, but dynamic *in-vivo* joint kinematics have been replicated [10, 16]. In position control, the relative translations are typically small (mm), and the angular velocity low (< 100 degrees/sec) [16]. If the desired loads at the joint are known, e.g. derived from inverse dynamics, force control can be employed. However, accurate force replication necessitates temporal scaling, leading to poor replication of load rate [17]. While joint simulators extend biomechanical testing beyond the confines of a UTM, the sub-physiologic speeds and load rates fail to reproduce the inertial environment necessary to investigate bone-prosthetic interface biomechanics. These limitations prevent full mechanical characterization of biologic systems where high-speed 3D kinematics are expected, such as percutaneous osseointegrated (OI) implant systems (Fig 1).

Percutaneous OI systems have the inherent risk of proximal bone fracture due to the direct skeletal endoprosthesis attachment. The cost of bone-implant interface fracture is severe, requiring a revision surgery or worse, loss of the entire limb [18]. Although fracture is typically associated with high energy trauma, transhumeral amputees could experience fracture as a result of loads generated during moderate demand activities [19, 20]. To mitigate this risk, percutaneous OI systems may be equipped with a compliant overload protection device to shield against excessive loading [18, 21]. Because bone fracture loads are direction dependent [22], strain rate dependent [23, 24] and load rate dependent [25, 26] it is desirable to mechanically characterize the bone-implant interface utilizing *in vivo* 3D kinematics.

UTMs are not designed to test the bone-implant interface or overload protection devices under the multiaxial, high-speed, dynamic motions experienced by the percutaneous OI systems *in vivo*. In contrast, force control of a robotic manipulator can mimic these physiologic loads. The presented method compliments this strategy in two ways. First, it replicates both the load and rate of load application, more closely mimicking physiologic loading conditions. Second, it enables testing of a prosthesis equipped with a compliant overload protection device colliding against a pliable obstacle (e.g. another human) where the load and load rate cannot be accurately predicted. Prior investigations have replicated *in vivo* kinematics of adjacent body segments using a robotic manipulator [10, 16, 27], and joint simulators can replicate global kinematics of the lower designed [28, 29], but no studies have replicated high-speed global kinematics of the upper extremity. This is challenging because of the high segment velocities and accelerations arising from the kinematic contributions of the lower extremity and torso.

Therefore, the purpose of this study was to create and validate a computational pipeline to replay high-speed, dynamic *in vivo* human kinematics on a robotic manipulator. Specifically, this study replicated humeral kinematics of advanced activities of daily living recorded via skin marker motion capture [19] on a 6 degree-of-freedom serial industrial manipulator. Since no fixed standards exist on replication accuracy (especially when considering velocity and acceleration), acceptable limits were derived from initial pilot data of replicating 3 jumping jack trials and the error contained in skin-marker derived glenohumeral kinematics. The pilot data indicated that 5% accuracy for position and 10% accuracy for velocity were attainable. Skin-marker derived glenohumeral kinematics contain 3–15% error when compared to kinematics derived from bone-pins [30]. Since error increases with successive differentiation, the goals of this study were set to replicate position/orientation to within 5% accuracy, velocity to within 10% accuracy, and acceleration to within 15% accuracy. The presented methodology can be applied to other human joint systems, extended to function on parallel manipulators, and/or utilized to improve upon existing joint simulators. Ultimately, the robotically replicated motions will be utilized in future investigations to mechanically characterize a percutaneous OI prosthesis system with a compliant overload protection mechanism.

## 2. Methods

### 2.1 Activities for robotic replication

Previously captured by Drew et al [19], the advanced activities of daily living (AADL) dataset consists of 120 trials (40 subjects, 3 trials/subject) for: jumping jacks, jug lift, jogging, rapid internal rotation, underhand toss, briefcase carry, and elbow fall (10.5281/zenodo.1040453). Briefly, torso, humerus, forearm, and hand trajectories were captured using a 10-camera optical motion analysis system (Vicon Motion Systems Ltd., Oxford, UK). Humeral motion was defined by a four-marker rigid cluster on a sleeve around the humerus and a single marker at the lateral epicondyle of the elbow. Marker trajectories were recorded at 200 Hz, gap filled, and low-pass filtered using a Butterworth filter with a cut-off frequency of 6 Hz. The humerus was modeled as a 6 degree-of-freedom rigid body in relation to the torso and its kinematics were derived from marker trajectories by utilizing Visual 3D (v5, C-Motion; Germantown, MD).

**Fig 2:**
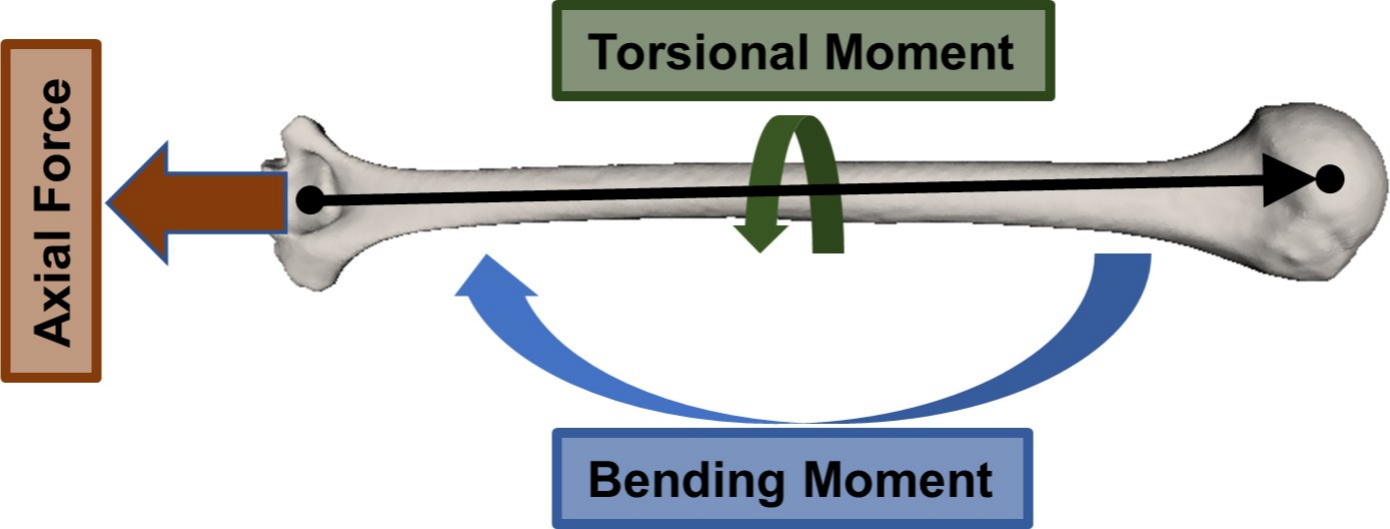
Schematic of the combined axial, bending and torsion loading modalities of the humerus. The humeral axis, as defined by the International Society of Biomechanics and in the AADL dataset, is the vector from the midpoint of the medial and lateral epicondyles pointing to the glenohumeral rotation center. Axial force is defined as the humeral reaction force projected onto the humeral axis vector; torsional moment, as the humeral reaction moment projected onto the humeral axis vector; and bending moment, as the humeral reaction moment projected onto the plane orthogonal to the humeral axis vector [19].

Two criteria determined whether an activity would be robotically replicated: 1) the activity’s utility in mechanically characterizing the bone-implant interface of a percutaneous OI system, and 2) its utility in determining the limits of the computational pipeline. Considering the characterization of the bone-implant interface, four of the seven activities were selected: jumping jacks generated high axial forces and high bending and torsional moments (Fig 2); jug lifts generated the highest sustained bending moments; jogging generated the highest axial forces; rapid internal rotations generated the highest torsional moments.

All 120 jumping jack and 120 jogging trials were included because they encompassed complex, high-speed angular and translational kinematics that test the limits of the computational pipeline. To properly simulate a jumping jack, high velocities and accelerations must be replicated within a relatively compact region of space. To simulate a jogging motion, high velocities and accelerations must be combined over a long distance. The jug lift and internal rotation trials were ranked within each activity according to the primary humeral kinematic variable affecting their kinetics, bending and torsional acceleration, respectively. The highest 5, middle 5, and lowest 5 trials yielding unique subjects for each motion were selected to represent the entire population. For jogging, the motion between heel strike and maximum flexion of the humerus relative to the torso encompassed the highest axial acceleration and was selected for replication. Since this region contained a high initial and final velocity, a 4^th^ degree constant-jerk (jerk is the derivative of acceleration) polynomial—for both position and orientation—was utilized to create an artificial ramp-up and slow-down period of 30 timepoints (0.15 s) for the trajectory. For all other activities, the entire motion was replicated for each chosen trial.

### 2.2 Rigid body and robot kinematics

The pose of a rigid body can be fully parameterized using 6 generalized coordinates that determine its position (p_1_,p_2_,p_3_) and orientation (φ_1_,φ_2_,φ_3_) [31, 32]. A 4x4 homogeneous transformation matrix (**T**) encompasses these parameters using a 3x3 rotation matrix **R**, and a 3x1 translation vector, **t**. This nomenclature will be utilized to denote the pose of a rigid body as measured in a reference frame, e.g. the humerus (H) in the lab (L). [32, 33]

$${}^{L}T_{H}=\left[\begin{array}{cccc} {}* & * & * & *\\ {}* & {}^{L}R_{H}(\phi_{1},\phi_{2},\phi_{3}) & * & {}^{L}t_{L\to H}(p_{1},p_{2},p_{3})\\ {}* & * & * & *\\ 0 & 0 & 0 & 1 \end{array}\right]$$ (1)

The transformation matrix can also map the pose of a rigid body from one reference frame to another, e.g. the humerus pose mapped from lab frame to the thorax (T) frame:

$${}^{T}T_{H}={\left({}^{L}T_{T}\right)}^{-1}∙{}^{L}T_{H}$$ (2)

The robot working envelope refers to the volume which can be reached by the end-effector, and is a function of its link lengths, joint configurations, and joint limits. The forward kinematics of the robot, and the physical limits of its joints, completely define the envelope and provide a mapping from the angular measurements of the robot (R) joints (***q***) to the pose of the end-effector (EE), where K is the robot forward kinematics function [32].

$${}^{R}T_{EE}=K(q)$$ (3)

### 2.3 Robotic, optical tracking, and motion capture systems

**Fig 3:**
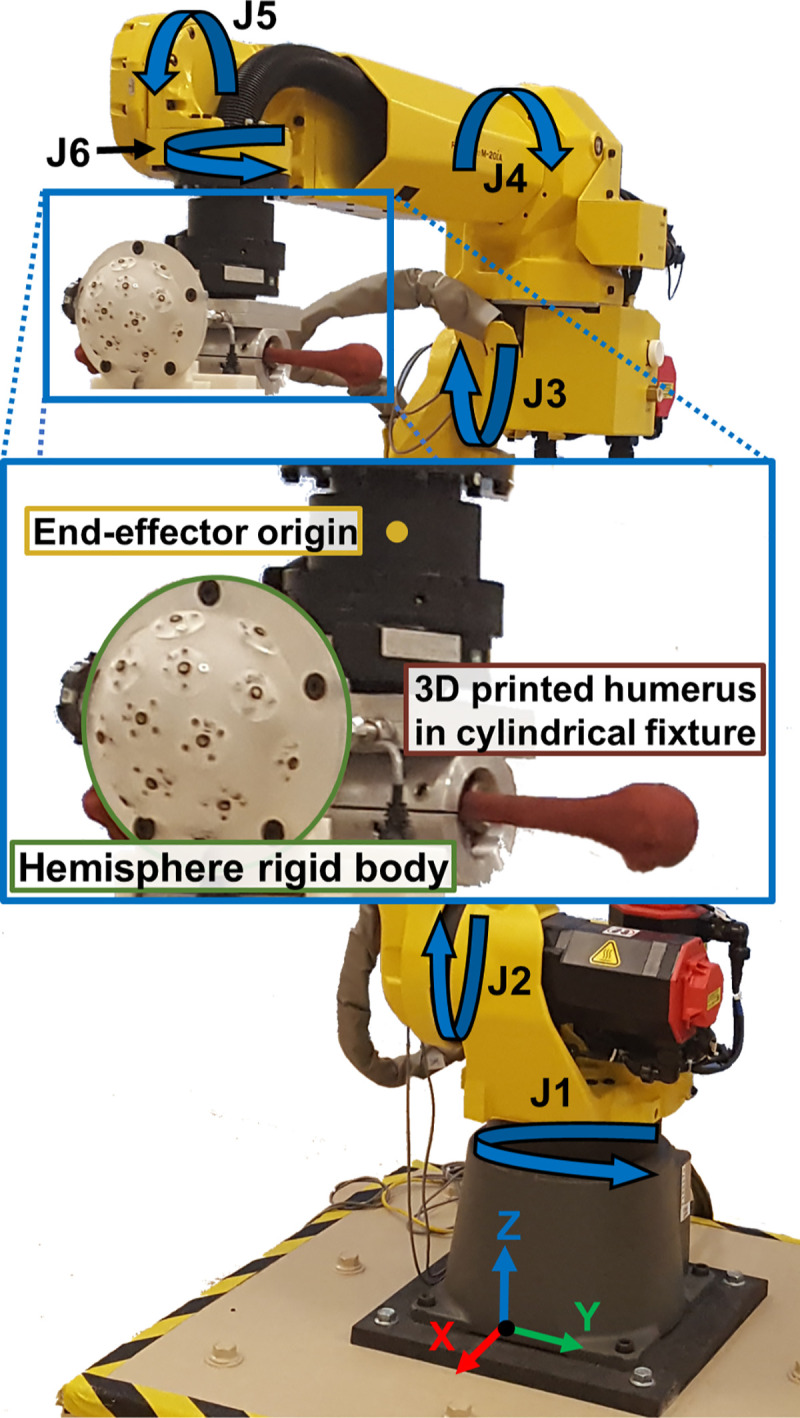
A 6 degree-of-freedom robot with optical tracking hemisphere rigid body and 3D printed humerus. The rotational motion of the robot’s joints (J1-J6) is indicated by the blue arrows, and the Cartesian coordinate system of the robot is shown at its base.

A 6-axis industrial robotic manipulator (Fig 3) was utilized to replicate humeral trajectories (M20iA, 20 kg payload, ±0.03 mm end-effector repeatability, FANUC America, Rochester Hills, MI). An optical tracking system verified robot positioning (Optotrak Certus, 0.1 mm accuracy, 0.01 mm resolution, Northern Digital Inc., Ontario, Canada). The Cartesian coordinate systems at the base of the robot, optical tracking system, and motion capture systems are referred to as the robot frame (R), optical tracking frame (OT), and motion capture frame (MC), respectively.

### 2.4 Robot and humerus frame identification

A custom hemisphere rigid body comprised of 16 infrared markers was rigidly mounted to the end-effector of the robot (Fig 3) and optical tracking software (6D Architect, Northern Digital Inc., Ontario, Canada) was used to define its Cartesian coordinate system. A humerus, 3D printed from a CT scan of a female arm, was rigidly mounted to the end-effector via a cylindrical fixture. The centroid of a sphere fit of 100 points on the articular surface of the humeral head, captured using the digitizing probe of the optical tracking system, defined the humeral head center. This landmark, along with the medial and lateral epicondyles, were used to establish a humeral (H) coordinate system in relationship to the hemisphere (HS), ^*HS*^***T***_*H*_, in accordance to the AADL dataset [19].

To program a humerus trajectory onto the robot, the rigid body relationship between the end-effector (EE) of the robot and the humerus (H), ^*EE*^***T***_*H*_, was necessary. This was computed from the rigid body relationship between the hemisphere and the humerus, and the end-effector and the hemisphere:

$${}^{EE}T_{H}={}^{EE}T_{HS}∙{}^{HS}T_{H}$$ (4)

To verify the trajectory replicated by the robot, a rotational transformation between the optical tracking and robot frame, ^*R*^***R***_*OT*_, was needed. The translation component of the transformation between the optical tracking and robot frame was superfluous (see Section 3.7). Since the optical tracking system was mobile relative to the robot, ^*R*^***R***_*OT*_ could change. Therefore, an automatic robot reference frame identification procedure for obtaining both ^*R*^***R***_*OT*_ and ^*EE*^***T***_*HS*_ (performed in under 2 minutes) was devised (see S1 Appendix [32, 34–36]). This was performed at the beginning of an experimental session, or if the optical tracking system was moved within a session.

### 2.5 Mapping of motion capture data to the robot joint space

The AADL dataset provided the rigid body trajectory of the humerus with respect to the motion capture frame, ${}^{MC}T_{H}^{k}$, where the right superscripted ‘k’ denotes the k^th^ timepoint. To program the humerus trajectory on the robot, it was necessary to map trajectories from the motion capture frame to the robot frame. This mapping is mutable–it can and should change from one trajectory to another. Also, in order to preserve the kinetic properties of the trajectories, this mapping could only be composed of a rotation about the gravitational axis and a 3-dimensional translation (4 parameters in total). Letting ***R***^***g***^(*θ*) represent a rotation about the gravitational axis by θ, and ^*R*^***t***_*MC*→*R*_ represent the 3-dimensional translation from the motion capture to the robot reference frame, the mapping can be expressed as:

$${}^{R}T_{MC}=[\begin{array}{cccc} {}* & * & * & *\\ {}* & {}^{R}R_{MC}^{g}(\theta ) & * & {}^{R}t_{MC\to R}\\ {}* & * & * & *\\ 0 & 0 & 0 & 1 \end{array}]$$ (5)

Eq (5) can be utilized to obtain the desired (indicated by a left subscripted ‘D’) trajectory of the humerus in the robot reference frame as shown in Eq (6):

$${}_{D}^{R}T_{H}^{k}={}^{R}T_{MC}∙{}^{MC}T_{H}^{k}\forall k$$ (6)

Utilizing the forward kinematics function of the robot and the rigid body relationship between the humerus and the robot end-effector, the humerus trajectory can subsequently be mapped to the robot joint space:

$$K(q(k))={}_{D}^{R}T_{H}^{k}∙{\left({}^{EE}T_{H}\right)}^{-1}$$ (7)

Eq (7) is the basis for two non-linear optimization problems that were devised to map trajectories from the motion capture frame to the robot joint space. Both problem formulations optimize the robot joint space trajectory so as to minimize the robot’s joint velocity utilization across the trajectory. Furthermore, both formulations utilize the four parameters of Eq (5), as well as the robot joint and joint velocity limits, as constraints. The first method (see S2 Appendix [31, 37–43]), termed the **Derivative-Free Optimization Algorithm**, relies on the constrained optimization by linear approximation (COBYLA) method [40] to optimize over the initial joint angles of the robot, ***q***(0). This formulation does not necessitate computing the gradient of the objective function or (in)equality constraints. The second method (see S3 Appendix [31, 37, 42–45]), termed the **Gradient-Based Optimization Algorithm**, employs the Sparse Nonlinear Optimizer (SNOPT) software package [44] which utilizes a sparse sequential quadratic programming algorithm to optimize over the entire joint space trajectory. This formulation does not require, but is more computationally efficient when, the gradient of the objective function and (in)equality constraints are computed.

Both algorithms optimized for the current pose of the humerus as attached to the robot, ^*EE*^***T***_*H*_, i.e. the current tool frame of the humerus [32, 46]. An additional 35 virtual tool frames were generated by programmatically rotating the humerus in 30° increments while clamped within the cylindrical fixture at 3 positions: ~1” distal to the humeral head, ~1” proximal to the midpoint of the epicondyles, and at midshaft. Each motion capture trial was optimized using each of the 36 tool frames. A tool frame was considered a successful match for a particular trial if the optimization converged and all constraints were satisfied. When robotically replicating a particular trajectory, an attempt to physically match the desired virtual tool frame was made by rotating and sliding the humerus within the cylindrical fixture. Since a perfect match was impossible, the humerus was digitized once more when clamped in the desired position and the optimization algorithm was re-run with the new physical humeral tool frame.

### 2.6 Program joint space trajectory onto robot

The humeral motion capture trajectory encompasses not only the pose of the humerus, but also its velocity and acceleration, both linear and angular. Industrial robots generally only specify the position, not the velocity or acceleration of the tool frame at a programmed point. Some control over the velocity and acceleration can be exerted by specifying a maximum velocity between two programmed points and a smoothness parameter [47]. The details of the smoothness parameter and its implementation vary by manufacturer, but generally specify a tradeoff between path versus velocity/acceleration accuracy (see S4 Appendix [47, 48]).

To implement the smoothness parameter, industrial robots utilize proprietary look-ahead features that allow the controller to evaluate future timepoints in the trajectory to compute joint torques that will produce the desired motion. However, the look-ahead feature can be disadvantageous because it limits the speed of the robot as it assumes that the final look-ahead point is the last timepoint in the trajectory. This is inconsequential with the sparse trajectories of traditional industrial applications, but not for dense trajectories of motion capture data (up to 200 Hz), where the resulting robot motion was much slower than desired.

To mitigate this issue, a non-uniform subsampling algorithm that took into account both position and orientation (see S5 Appendix [36, 49]) was developed. All trajectories were subsampled to keep only 20% of the original datapoints. The percentage was determined based on robot manufacturer recommendation that programmed points should be approximately 24 ms apart. Since the motion capture data sampling period was 5 ms, a subsampling percentage of 20% corresponded to points spaced 25 ms apart on average.

### 2.7 Verify robot motion

Each selected humeral trajectory was mapped from the motion capture reference frame to the joint (and operational) space of the robot via the optimization algorithms (Section 3.5) to obtain ^*R*^***T***_*MC*_. The trajectories were then subsampled and programmed onto the robot (Section 3.6). The robot frame identification procedure (Section 3.4) was executed at the beginning of each experimental session to obtain the rotational transformation between the optical tracking and robot frame, ^*R*^***R***_*OT*_. The robot program was run and the pose trajectory of the hemisphere was recorded by the optical tracking system: ${}^{OT}T_{HS}^{1},\ldots ,{}^{OT}T_{HS}^{n}$ where n denotes the total number of timepoints. The humerus pose in the robot reference frame was obtained per Eqs (8) and (9). The left subscripted ‘A’ denotes the trajectory achieved by the robot, and the right superscripted ‘j’ denotes the j^th^ timepoint.

$${}^{R}T_{OT}=[\begin{array}{cccc} {}* & * & * & 0\\ {}* & {}^{R}R_{OT} & * & 0\\ {}* & * & * & 0\\ 0 & 0 & 0 & 1 \end{array}]$$ (8)

$${}_{A}^{R}T_{H}^{j}={}^{R}R_{OT}∙{}^{OT}T_{HS}^{j}∙{}^{HS}T_{H}\forall j$$ (9)

For ease of interpretation both the desired and actual translations were normalized based off the first waypoint in the trajectory. Translating every timepoint in a trajectory by the same amount does not affect the kinetic properties of the trajectory, hence Eqs (10) and (11) are justified.

$${}_{D}^{R}t_{H}^{k}={}_{D}^{R}t_{H}^{k}-{}_{D}^{R}t_{H}^{1}\;\forall k$$ (10)

$${}_{A}^{R}t_{H}^{j}={}_{A}^{R}t_{H}^{j}-{}_{A}^{R}t_{H}^{1}\;\forall j$$ (11)

**Fig 4:**
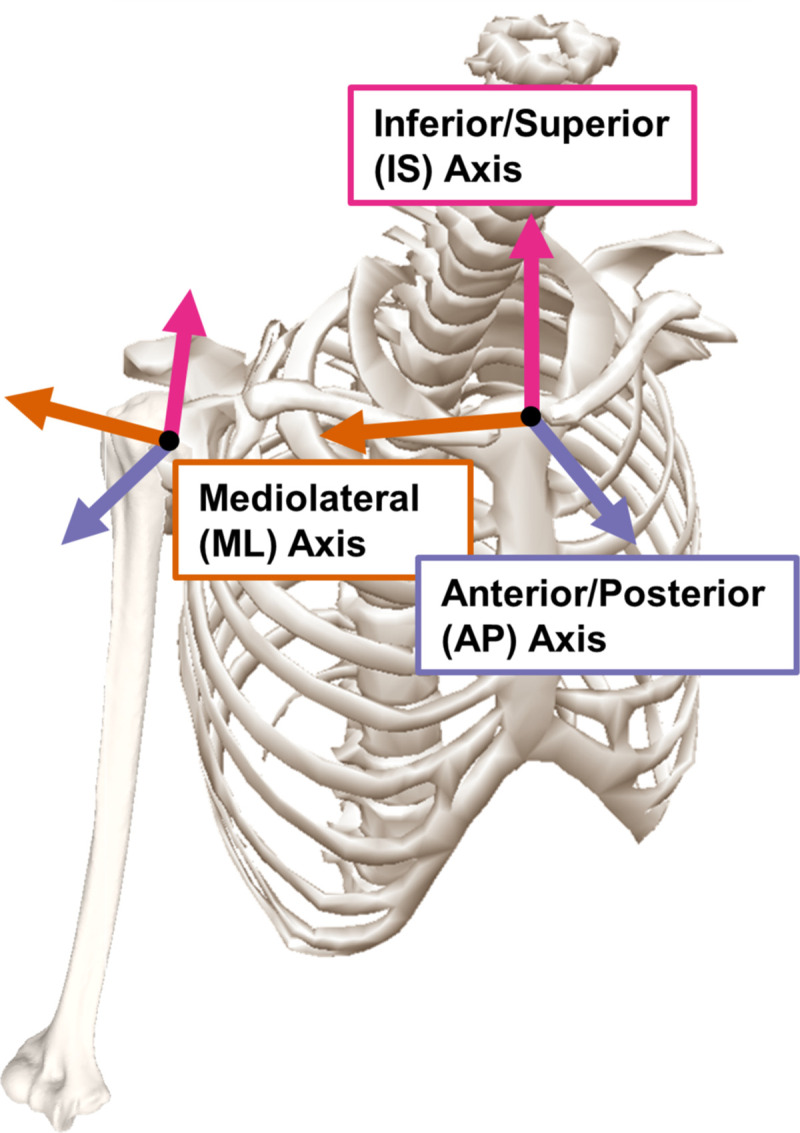
Thorax anatomical axes. The thorax coordinate system was the reference coordinate system for all presented results. This system was defined in the input motion capture data and was unique to each subject in the data set based on their anthropometrics and associated locations of skin markers. The humerus coordinate system is defined to coincide with the thorax coordinate system when in neutral anatomical position [51]. The thorax bone model was reprinted under a CC BY license, with permission from C-Motion Research Biomechanics.

Subsequently, all orientations were converted to the rotation vector formulation [31]. The achieved pose trajectory of the humerus was smoothed using a 8Hz fourth-order bidirectional Butterworth filter, then differentiated once to obtain linear and angular velocity [43], and a second time to obtain linear and angular acceleration. The achieved pose, velocity, and acceleration of the humerus as actuated by the robot and tracked via the optical tracking system was compared against the desired pose, velocity, and acceleration of the humerus as derived from the AADL motion capture dataset [19]. Trajectories were temporally aligned by determining the offset that produced the maximum cross-correlation between the desired and achieved position and rotation vectors simultaneously. The pose of the thorax in the initial frame of a trial was utilized as an intuitive reference frame for all metrics. The normalized mean absolute error (MAE) between the achieved and desired humeral trajectories for pose, velocity, and acceleration was computed for each trial for each anatomical axes of the thorax (Fig 4). Likewise, the normalized MAE between the achieved and desired humeral trajectories for the magnitude of pose, velocity, and acceleration was computed for each trial. For all kinematic variables the magnitude was computed by utilizing Euclidean distance. The normalization factor was the corresponding span of the desired (motion capture) data, e.g. the MAE of the linear velocity in the inferior/superior direction was normalized by the span (maximum-minimum) of the desired linear velocity in the inferior/superior direction [50].

### 2.8 Comparison of desired and actual kinetics

The desired and achieved humeral kinematics were utilized to calculate the loads (forces and moments) at the 25% amputation level of the humerus via inverse dynamics. The 25% amputation level was chosen since the loads were highest due to the increased moment arm distal to the resection [19, 52]. The prosthesis was modeled as a rigid body with the hand, forearm, and the connection from the forearm to the residual bone each modeled as a cylinder (Table 1). This simple model allowed comparison of the loads generated from the kinematics derived from skin markers (desired) and reproduced via the robot (achieved). The elbow was set to 90° flexion for jogging and internal rotation motions, 135° for jumping jacks, and 180° extension for jug lift motions. Standard inverse dynamics methods (recursive Newton-Euler algorithm [37]) were utilized to obtain the loads. Normalized MAE was computed for the axial force, bending moment and torsional moment of jumping jacks, bending moment of jug lifts, axial force of jogging, and torsional moment of internal rotation trials. In addition, the normalized absolute value of the difference between the desired and achieved load at the instance when the maximum desired load is achieved, termed normalized error-at-peak, was calculated. Differentiation and the composition of noisy signals via mathematical operations (inverse dynamics) led to spikes for both the achieved and desired trajectories that are more likely a result of noise than a true representation of the human motion [53]. The error-at-peak metric reduced (although did not eliminate) the effect of the increased noise level.

**Table 1:** Dimensions and masses of the segments constituting the prosthesis system model utilized for inverse dynamics.

Segment/Measurement	Length (m)	Diameter (m)	Mass (kg)
**Arm Connection**	0.75 * Arm Length	0.060	0.50823 (kg/m) * 0.75 * Arm Length + 0.3
**Forearm**	0.2540	0.0762	1.013
**Hand**	0.1524	0.0540	0.416
**Jug**	0.2500	0.1500	3.800

Note: The Arm Length variable was derived from the motion capture dataset and is dependent on the anthropometrics of the subject.

### 2.9 Code repositories

The following repositories were created for the software and the supporting dataset:

- The derivative-free optimization algorithm: https://doi.org/10.5281/zenodo.3665788
- The gradient based optimization algorithm: https://doi.org/10.5281/zenodo.3665786
- The subset of trials from the AADL dataset pertinent to this investigation, as well as the robot verification data from the optical tracking system: https://doi.org/10.5281/zenodo.3661595
- Remainder: https://doi.org/10.5281/zenodo.3665790, https://doi.org/10.5281/zenodo.3665780. These repositories include the following procedures and algorithms:
  ○ Artificial ramp-up and slow-down trajectory generation
  ○ Robot reference frame identification procedure
  ○ Non-uniform subsampling algorithm
  ○ FANUC motion program generator
  ○ Comparison of robotically replicated trajectories against motion capture trajectories

## 3. Results

### 3.1 Mapping of motion capture data to the robot joint space

In practice, the derivative-free optimization proved to be more effective than the gradient-based optimization for initial trajectory optimization. It consistently converged to an optimal solution even when the initial seed was simply the robot home position. The gradient-based optimization was useful when the virtual humeral tool frame was substituted with a physical one. In this instance, given a joint space trajectory that was optimal for the virtual tool frame, the gradient-based optimization quickly determined a joint space trajectory that was optimal for the physical tool frame.

The optimization algorithms found suitable joint space trajectories for 119 jumping jacks (1 was omitted due to motion capture artefacts), and all 15 jug lifts. A suitable joint space trajectory that satisfied the robot joint velocity limits for the internal rotation trial with the single highest torsional acceleration could not be found. However, a joint space trajectory for the next 5 internal rotation trials with the highest torsional accelerations was found, as well as for the middle 5 and lowest 5 trials. Six jogging trials were excluded because the section of the trial between heel strike and maximum flexion of the humerus was not present in the capture. One jogging trial was excluded due to motion capture artefacts. Out of the remaining 113 jogging trials, a suitable joint space trajectory was found for 105 trials but 8 trials could not satisfy the robot joint velocity limits. All 119 jumping jacks, 15 internal rotations, and 15 jug lifts were replicated using a single humeral tool frame. Jogging trials were replicated using 4 humeral tool frames: 65 were replicated utilizing the same tool frame as other motions, 20 in a tool frame rotated ~60° about the humeral axis, 17 rotated ~120°, and 3 rotated ~150°.

### 3.2 Verify robot kinematics

**Fig 5:**
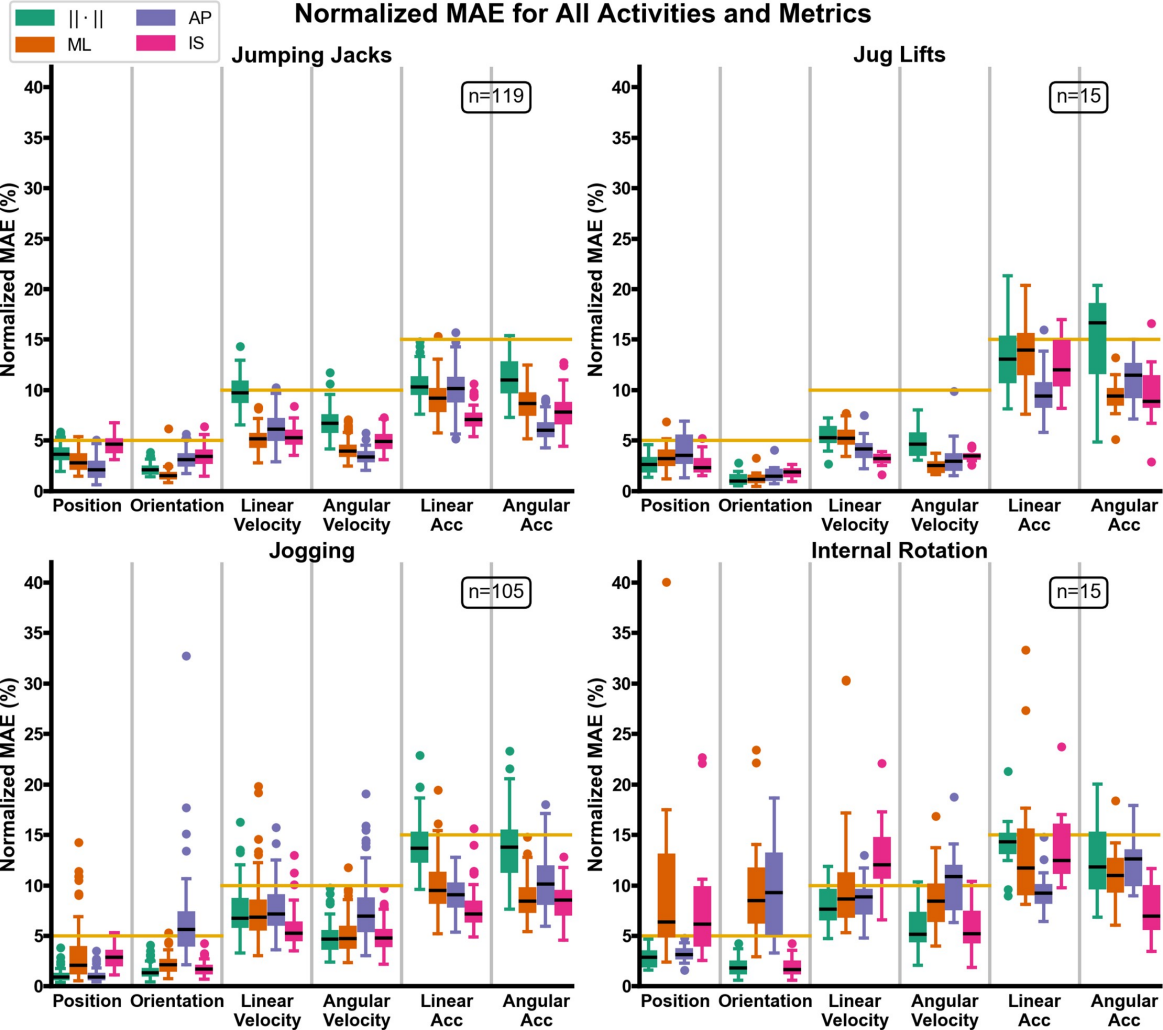
Boxplots of normalized MAE for all activities and metrics. For each activity and kinematic variable, the normalized MAE in the mediolateral (ML), anterior-posterior (AP), and inferior-superior (IS) direction of the thorax is presented. The normalized MAE of the magnitude of each kinematic variable is also presented and denoted via the Euclidean norm (‖∙‖). Gold bars represent the cutoff values proposed as acceptable limits of robotic simulation error.

**Fig 6:**
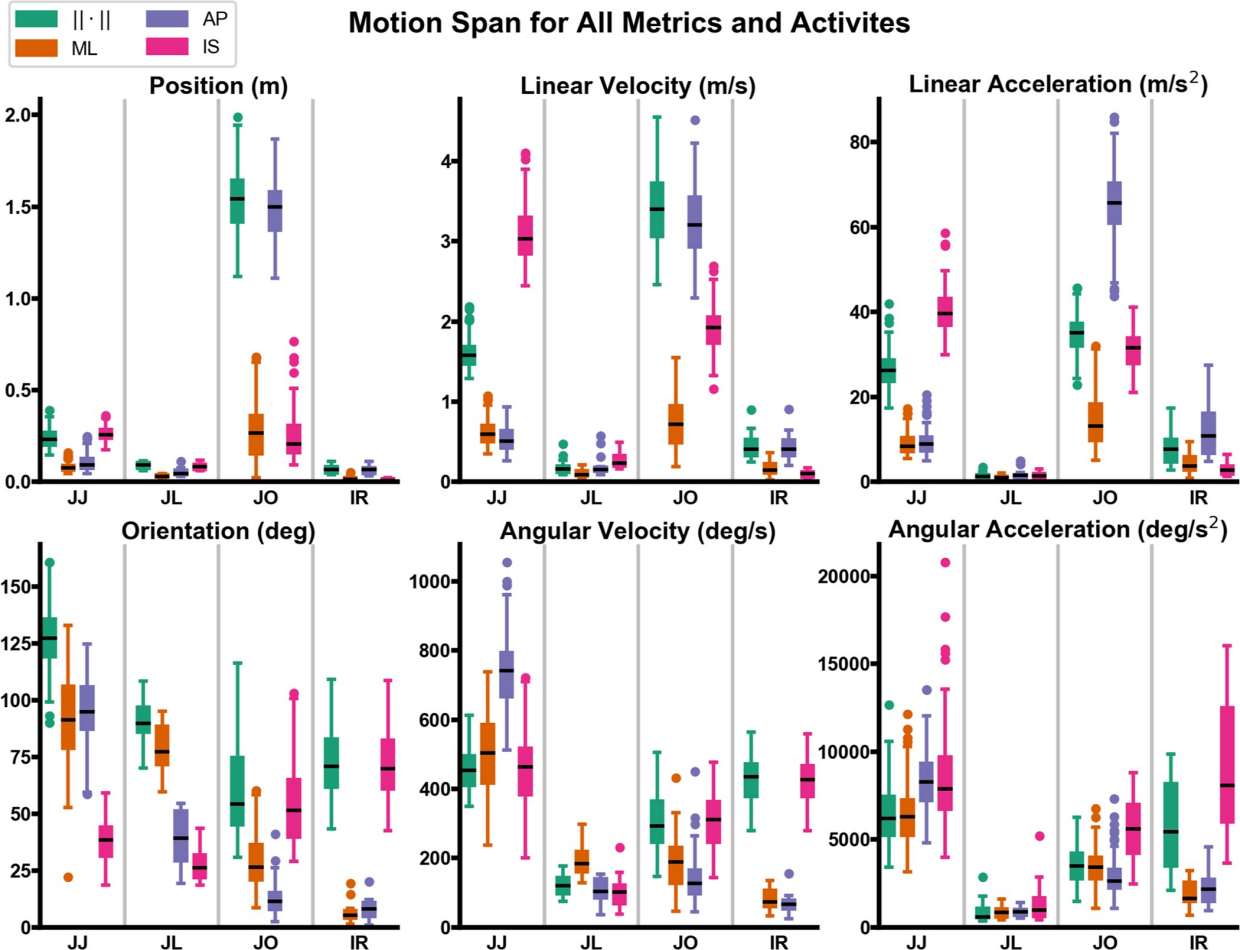
Boxplots of motion span for all activities and metrics. For jumping jacks (JJ), jug lifts (JL), jogging (JO), and internal rotation (IR) trials the span (max-min) of each kinematic variable in the mediolateral (ML), anterior-posterior (AP), and inferior-superior (IS) direction of the thorax is presented. The span of the magnitude of each kinematic variable is also presented as the Euclidean norm (‖∙‖).

For all activities, the median pose normalized MAE was smaller than the median velocity normalized MAE, which was smaller than the median acceleration normalized MAE. For all activities, the median normalized MAE of the position and orientation magnitude was under 4%, and normalized MAE of the position and orientation magnitude for all trials was under 6%. For all activities, the median normalized MAE of the linear and angular velocity magnitude was under 10%. The normalized MAE of the linear and angular velocity magnitude for all trials for jumping jacks, jug lifts, and internal rotation was under 15%, while for jogging was under 17%. The median normalized MAE of the linear and angular acceleration magnitude was under 15% for jumping jacks, jogging, and internal rotation, while for jug lifts it was under 17%. The normalized MAE of the linear and angular acceleration magnitude for all jumping jack trials was under 16%, and was under 24% for all jug lifts, jogging and internal rotation trials (Fig 5). The normalization factors for all activities, metrics, and anatomical axes are presented in Fig 6.

Trend plots for desired versus achieved pose, velocity and acceleration associate a visual representation of performance with a normalized MAE value for a trial representative of median performance for each activity and kinematic variable (S6 Appendix). A video of the robot replicating a jumping jack and a jogging motion are also presented (S1 and S2 Videos, respectively).

### 3.3 Kinetic analysis

**Fig 7:**
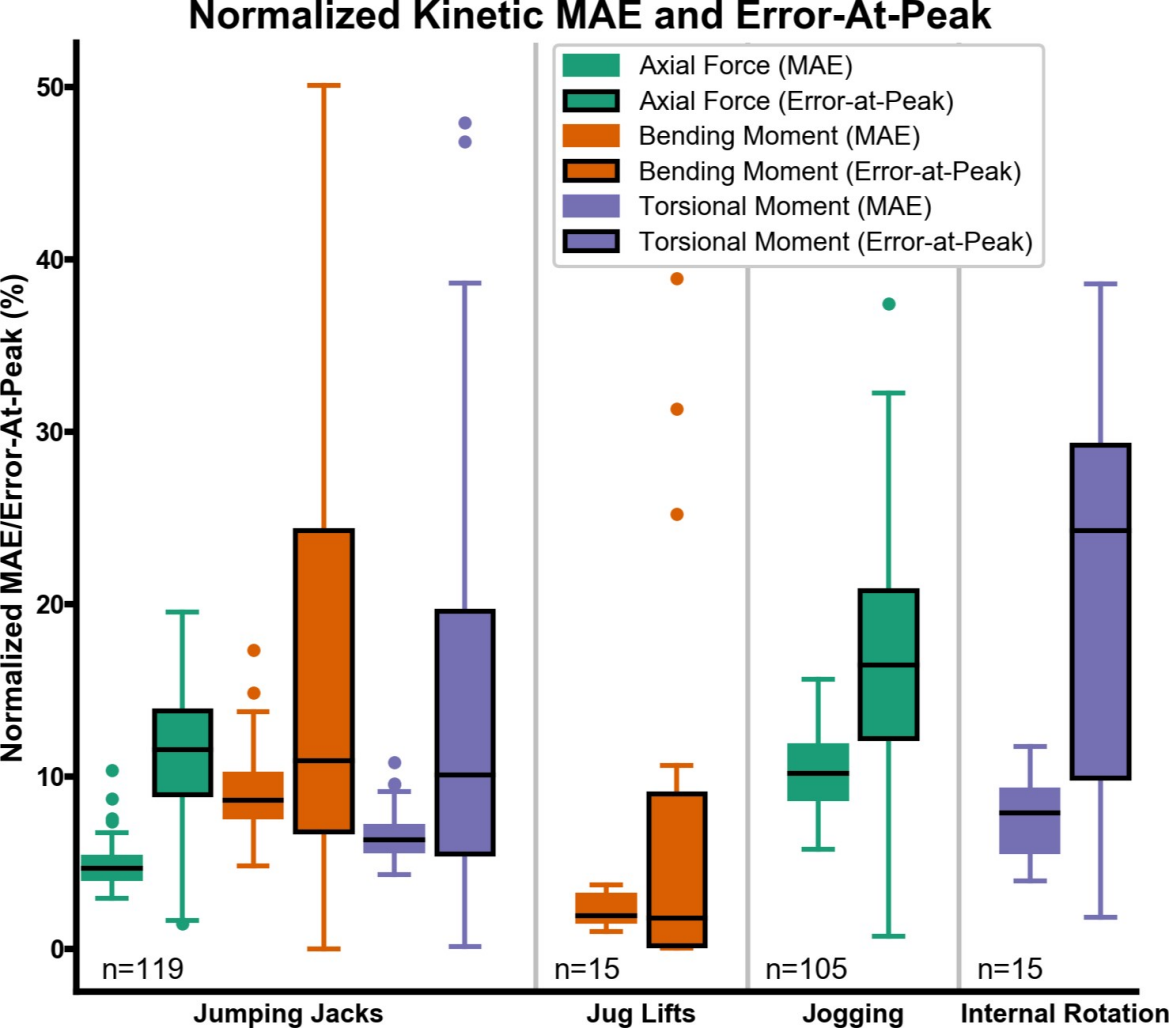
Boxplots of normalized kinetic MAE and error-at-peak. The relevant kinetic variables are grouped by activity. Error-at-peak is represented by a solid line around the box.

**Fig 8:**
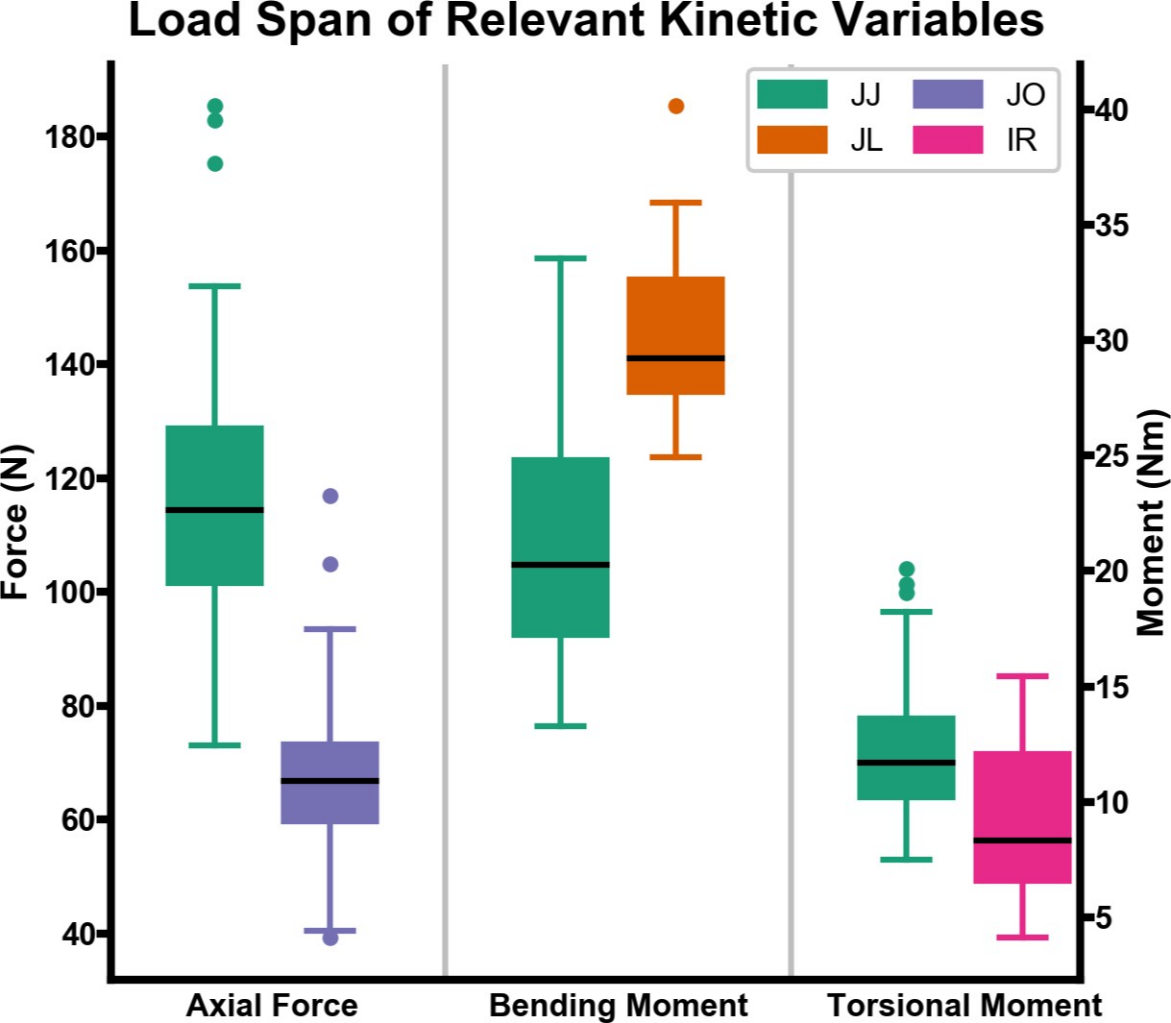
Boxplots of load span for axial force, bending moment, and torsional moment. For jumping jacks (JJ), jug lifts (JL), jogging (JO), and internal rotation (IR) trials the span (max-min) of the applicable kinetic variable is presented.

The median normalized MAE for all activities and variables was under 10%, and the normalized MAE for all activities and variables was under 20%. The median normalized error-at-peak was under 12% for jumping jacks, 2% for jug lifts, 17% for jogging, and under 25% for internal rotation. There was large variability in the highest normalized error-at-peak, ranging from 11% for the bending moment of jug lifts up to 50% for the bending moment of jumping jacks (Fig 7). The normalization factors for axial forces, bending moment, and torsional moment for all activities are presented in Fig 8.

## 4. Discussion

The primary goal of this investigation was to robotically replicate *in vivo* humeral kinematics of jumping jacks, jug lifts, jogging and internal rotation advanced activities of daily living on an industrial manipulator to within 5% accuracy for position and orientation, 10% accuracy for linear and angular velocity, and 15% accuracy for linear and angular acceleration. An equally important objective was to provide a computational pipeline for mapping *in vivo* kinematics recorded via motion capture to a joint space trajectory for a robotic manipulator via public code repositories (Section 3.9). The median normalized MAE for the magnitude of all kinematic variables was generally within targeted accuracy, with the exception of angular acceleration for jug lifts (17%). Considering each anatomical axis individually, the target accuracy goal was achieved for the median normalized MAE for all kinematic variables and across all activities with the following exceptions. For jogging, the median accuracy goal was not achieved for the anterior/posterior (AP) axis of orientation (5.6%). For internal rotation, the median accuracy goal was not achieved for the mediolateral (ML, 6.4%) and inferior/superior (IS, 6.2%) axes of position, the ML (8.5%) and AP axes (9.3%) of orientation, the IS axis of linear velocity (12.1%), and the AP axis of angular velocity (10.9%). Despite not meeting all accuracy goals, the robotically replicated trials are kinematically accurate to within the characteristic error contained in skin-marker derived kinematics. These robotically replicated motions improve upon UTM capabilities by providing multiaxial kinematics, and in contrast to a typical joint simulator, allow researchers to more closely replicate the load rates experienced by the humerus while permitting experiments into failure scenarios like collisions. Furthermore, the methods presented herein provide an algorithmic pipeline for other joint simulators and a starting point for future investigations to improve upon these results.

Although jug lifts had the lowest median angular acceleration of any activity (Fig 6), they had the highest normalized MAE for the angular acceleration magnitude. This apparent anomaly is likely explained by their lower velocity and consequently lower signal-to-noise ratio as compared to other activities. Even though the motion capture trajectories were smoothed, double differentiation sharply increased the noise component, overwhelming the underlying motion signal for jug lifts trials. This was especially evident in angular kinematic variables, for which noise levels were inherently higher. Visual inspection of the trend plots for the kinematic variables of a representative jug lift trial (S6 Appendix) supports this interpretation. Therefore, the replication accuracy goal was not met due to the lower signal-to-noise levels of these trials rather than the capability of the computational pipeline or the robot. In the context of mechanically characterizing the bone-implant interface, the higher error associated with the magnitude of angular acceleration for jug lifts is not concerning because over 90% of the bone bending moment is generated by the gravitational force of the jug rather than inertial forces.

Investigation into the missed accuracy goals for jogging and internal rotation trials shows that the MAE normalization factor, the span of the trajectory, was the culprit. For jogging and internal rotation trials, the kinematic variable/anatomical axis combinations for which the motion span approaches zero are precisely the combinations that display the most variability in the normalized MAE and/or fail to meet the target accuracy goals (Figs 5 and 6). For jogging, the position span for the ML axis varies between 24–681 mm (Fig 6). This anatomical axis displays the greatest variability for the position normalized MAE, although on average it does meet the target accuracy goal. Likewise, for jogging the orientation span for the AP axis varies between 2.5°-41°. AP is the anatomical axis that displays the most variability for the orientation normalized MAE and misses the target accuracy goal. For internal rotation trials, the position span varies between 1–52 mm for the ML axis and between 1.5–23 mm for the IS axis. Likewise, for internal rotation the orientation span varies between 1.3°-19.4° for the ML axis and 0.9°-20.1° for the AP axis. Again, these anatomical axes display the most variability for the orientation normalized MAE and fail to meet the target accuracy goal. The same analysis carries over to the velocity kinematic variables for internal rotation trials.

For jogging and internal rotation trials, none of the kinematic variable/anatomical axis combinations for which the accuracy goal was missed were the primary axes of motion. The normalization factor for the MAE metric is induced by the reference frame selected for analysis (thorax pose in the initial frame). This reference frame, especially for internal rotation trials, magnifies errors associated with secondary axes of motion. Therefore, the higher than desired normalized MAE were deemed acceptable in the context of this study and within the characteristic error contained in skin-marker derived kinematics. Both objectively and subjectively (trend plots) the lowest replication accuracy was observed for internal rotation trials–for which the error associated with skin-marker derived kinematics is much higher (10–15% versus 3–15% [30]). Lastly, other authors have under scaled *in vivo* kinematics before robotic replication based on the fact that skin-marker kinematics overestimate range of motion [16]. This approach was not taken in the present investigation because there is no validation that the resulting kinematic trajectories are more accurate than the original motion capture trajectories.

The normalized error-at-peak metric demonstrates that only a subset of the robotically replicated trials should be used for biomechanical investigations, depending on the desired level of accuracy in recreating maximal loading conditions. Although there is wide variability for error-at-peak, 50% of the trials have a normalized value below 12% for jumping jacks, 2% for jug lifts, and 17% for jogging (Fig 7). This is encouraging because it demonstrates that there are still many trials in the dataset that are likely to accurately recreate the maximal loading conditions at the bone-implant interface. As previously noted, part of the variability in the error-at-peak stems from the noise in the signal. Although both the desired and actual raw signals have been filtered, the optimum filtering frequency varies based on the target metric (velocity, acceleration, force, etc.) [54] and does not account for soft tissue artefact [55]. These signal-processing challenges are demonstrated by the bending moment of jug lifts (Fig 7), which is almost entirely dependent on the orientation of the prosthesis (and jug) with respect to gravity. Sudden spikes in angular velocity and acceleration cause increased bending moments, introducing the outliers observed for the bending moment of jug lifts.

Increased noise only contributes partially to the MAE and error-at-peak, since robot limitations have large contributions as well. Analysis of error-at-peak versus maximum desired load resulted in a coefficient of determination (R^2^) of 0.51, 0.41, and 0.84 for jumping jacks axial force, jogging axial force, and internal rotation torsional moment, respectively. All other variables showed a R^2^ < 0.2. Moreover, for internal rotation trials, the average normalized torsional moment error-at-peak for trials with the lowest desired torsional acceleration was 8.5%, while those with the highest desired acceleration was 32.3%. However, the optimization algorithm converged to a solution that satisfied the joint velocity limit limitations for all analyzed trials. Therefore, it is very likely that other undisclosed limitations (whether physical or at the controller level) exist within the robot. The accuracy of the internal rotation trials could possibly be improved by utilizing a fixture that changes the rigid body relationship between the humerus and end-effector so as to increase the utilization of the sixth joint of the robot (J6). J6 has the highest joint velocity limit, and we surmise may also have the highest joint acceleration limit. Such an investigation is beyond the scope of the present work because it is not generalizable. If the accuracy of internal rotation trials cannot be improved, only the low and a subset of the medium internal rotation trials will be utilized for mechanical testing.

Nevertheless, this investigation presents a method for replicating the physiologic conditions at the bone-implant interface that mimics the load directionality, magnitude, and rate to within reasonable error. The replication of kinematics can be used as a standalone test methodology or be paired with a force-controlled manipulator that more accurately replicates load magnitude and direction. Although a prosthesis was not attached to the robot end-effector during this validation study, we do not foresee that it would appreciably affect the robotically replicated trajectories. The mass of a typical above-elbow prosthesis is ~1.8 kg, which increases to ~5.6 kg when holding a gallon jug, so both fall well within the 20 kg maximum load capacity for the robot. Load rate was not examined quantitatively in this investigation because it would entail a third differentiation, greatly amplifying the noise in the original signal. However, qualitative analysis of trend plots, and the fact that the replicated activities are on the same time scale as the original motion capture, indicate an acceptable replication of load rate. This in contrast to other investigations that temporally scale *in vivo* kinematics by a factor of 4 to 50 [14, 17, 28, 56], even for relatively slow tasks such as walking.

Overall, this investigation demonstrates the feasibility of utilizing an industrial robot as a next generation UTM. These trials provide a motion library for investigations of bone and prosthesis biomechanics, which will be instrumental in mechanically characterizing percutaneous OI prosthesis systems by replicating the dynamic multiaxial kinematic environment that the bone-implant interface experiences during AADLs. The AADL motions selected for robotic replication were derived from skin-markers because (to the authors’ knowledge) kinematic recordings of high-speed, upper-extremity activities from more accurate motion capture technologies do not exist. Upper extremity kinematics datasets from biplane fluoroscopy [57, 58] or bone-pins [59, 60] are limited to slower movements and small capture volumes. Regardless, all algorithms presented in this study are motion capture technology agnostic. The same algorithmic pipeline can be utilized with segment kinematics obtained from any motion capture input.

The robot reference frame identification procedure provides a quick (<2 minutes), automatic method of obtaining a coordinate system transformation between an optical tracking system and a robotic manipulator. It also determines the pose of a rigid body (e.g. humerus) in the robot frame of reference. Although a seemingly simple task, determining the relationship between the robot end-effector and an attached segment is complicated by the fact that the end-effector reference frame cannot be visualized. For many joint simulators, segment coordinate systems are determined by neutral alignment assessed via measured forces or approximate visual alignment [2–4, 7]. Other studies attach metal rods and pipe fixtures along segment anatomical axes, which are then visually aligned against the robot frame axes [16, 61]. Van Arkel et al. acknowledge the general lack of detail in the literature regarding the establishment of a segment coordinate system and present a method for aligning the femur and pelvis against the robot frame axes by utilizing custom fixtures [62]. Although likely more accurate than other manual methods, this procedure still results in up to 4° of misalignment, necessitates custom fixtures, and still must be extended to other joints [62]. In contrast, the robot reference frame identification procedure presented herein can be applied to any segment of interest and has precision of less than 1° and 1 mm.

A similar procedure to our investigation was employed by El Daou et al. [46] to ascertain the relationship between the femur and a robot end-effector. Both utilized an optically tracked intermediate rigid body to determine the end-effector/bone relationship, but El Daou et al. employed a nonlinear parameter estimation solution. Unfortunately, the authors did not provide details regarding the algorithm nor present data on its precision. The code and associated data for executing, visualizing, and analyzing our algorithm are available with the hope that future investigations can compare several algorithms to establish the efficacy of each one. Future studies should also investigate how to establish the segment coordinate system with respect to the robot end-effector from medical image data. This enhancement could be implemented by using a secondary rigid body attached to the bone of interest, and would permit investigations that maintain the soft tissue structures associated with a segment.

Although the algorithms presented herein can be used with any motion capture system, the method by which industrial robots are programmed must be modified in order to replicate *in vivo* kinematics to the same accuracy as these technologies. Other studies have replicated *in vivo* kinematics on a joint simulator [10, 16, 27], but to the authors’ knowledge no investigation has compared the velocity and acceleration of the replicated motions (achieved) against the input kinematics (desired). As our results demonstrate, the disparity increases with each successive differentiation between the replicated and motion capture kinematics (Fig 5). From a signal-processing perspective, the increase in MAE with each successive differentiation is expected [63]. However, most of the discrepancy, especially for velocity and acceleration, can be attributed to the lack of robotic control over these metrics.

Current programming methods for almost all industrial robots sacrifice positional accuracy for velocity/acceleration accuracy (see Section 3.6) [47]. In a typical industrial robot application, the motion program is composed of waypoints at/near physical features of the object being manipulated. The speed of the robotic tool can be specified between these waypoints, however it is not critical to arrive at these waypoints with a particular velocity (unless a complete stop is desired). Consequently, the velocity at a waypoint cannot be specified. Yet when robotically recreating *in vivo* kinematics, it is desirable to specify not just the pose but also the velocity and acceleration at waypoints. It is also desirable to input all timepoints in a motion capture trajectory so that the robot controller can plan the corresponding motor torques. To the authors’ knowledge, this elevated level of robot controller access is not provided by the 4 major industrial robot manufacturers (KUKA, Yaskawa Motoman, ABB, FANUC). Until it is implemented by manufacturers, similar velocity and acceleration errors to the ones presented here should be expected.

The need for increased control and ease of programming is widely recognized. ROS-Industrial [64] is an open source project developed and maintained by the ROS-Industrial consortium as an add-on to the popular Robot Operatic System (ROS) [65]. They recognize the need for increased control and ease of programming for tasks other than “welding, material handling, and dispensing” [66]. In fact, the FANUC ROS-Industrial software driver faces the same limitations as our study: “dense trajectories … cause significant slowdown of the robot” while “coarse trajectories lead to inaccurate motions” [67]. Thus, the need to balance accuracy versus velocity led to the development of non-uniform subsampling to reduce the maximum error due to subsampling and replicate the curvature of the original trajectory more faithfully than uniform subsampling (see S5 Appendix). Ideally the need for this algorithm will diminish as increased access to the motion planning modules is provided by industrial robot manufacturers.

The presented algorithms for mapping a rigid body trajectory to a robot joint space trajectory were purposefully written for ROS in anticipation of this increased access. One advantage of the derivative-free optimization algorithm is its relative insensitivity to the number of timepoints in the capture because its optimization parameters are comprised of the robot joint angles in the starting pose (e.g. optimization parameter space is 6-dimensional since a 6 degree-of-freedom robot was utilized). Another key advantage is the ability to determine *a priori* whether a given trajectory can be replicated on a robot given its physical limitations. In this investigation, the robot joint velocity limits were known and utilized as inequality constraints in the non-linear optimization problem. However, other constraints such as joint torques and acceleration limits can be easily appended if known. Likewise, the optimization algorithms can determine the feasibility and utility of various tool frames for the segment of interest. For example, by utilizing the derivative-free optimization algorithm, it was determined programmatically that 4 humerus tool frames were necessary to replicate jogging trials.

Even if it were possible to robotically replicate a given kinematic trajectory with complete accuracy, segment trajectories derived via skin-marker motion capture are subject to soft-tissue artefact (STA). STA attenuation is challenging [55, 68] as it has the same frequency content as the underlying bone motion [68], a large component of STA is rigid body transformations [69, 70], and skin marker displacements are subject, task and location specific [70]. Although it is possible to use different imaging modalities like biplane fluoroscopy [57, 58] or bone pins [59, 60] to derive kinematics, these techniques are difficult to implement for most studies because they are invasive, time consuming, expose patients to radiation, and require highly specialized equipment. Therefore, further investigation in quantifying and attenuating STA is necessary to increase the accuracy of robotically replicated human motion.

In conclusion, this investigation replicated *in vivo* humeral kinematics of jumping jacks, jug lifts, jogging and internal rotation activities using an industrial manipulator to within the accuracy of the skin-marker motion capture technology that was utilized to derive these kinematics. The replicated motions will be utilized to characterize the bone-implant interface of a percutaneous osseointegrated prosthesis system, but have numerous biomechanical applications. Several optimization and programmatic algorithms were developed, presented, verified and shared via public code repositories. In particular, an algorithm for identifying the robot reference frame and ascertaining the pose of a segment of interest via optical tracking motion capture was presented. This procedure improves upon current methods and provides utility to investigations employing joint simulators. Furthermore, a derivative-free and gradient based optimization algorithm for mapping motion capture trajectories to the robot joint space were developed as packages for the Robot Operating System. These algorithms will serve as a stepping stone in building the next-generation robotic universal testing machine that enables biomechanics researchers to investigate bone and prosthesis biomechanics by utilizing *in vivo* kinematics.

## Supporting information

S1 Appendix Robot reference frame identification procedure. (DOCX)

S2 Appendix Derivative-free optimization algorithm. (DOCX)

S3 Appendix Gradient based optimization algorithm. (DOCX)

S4 Appendix Programming optimized humeral trajectories on M20ia robot. (DOCX)

S5 Appendix Non-uniform subsampling algorithm. (DOCX)

S6 Appendix Representative trend plots for all activities. (DOCX)

S1 Video Video of robot replicating a jumping jack. (MP4)

S2 Video Video of robot replicating a jogging motion. (MP4)
