# Supplementary material for: Replicating dynamic humerus motion using an industrial robot
Source: PLoS One. 2020 Nov 9;15(11):e0242005. doi: 10.1371/journal.pone.0242005 (PMC7652298; doi:10.1371/journal.pone.0242005)
Supplement: S1 Appendix — (DOCX) [file pone.0242005.s001.docx]

**S1 Appendix - Robot Reference Frame Identification Procedure**

1. **Procedure Details**

Robot reference frame identification is a two stage process that calculates the rotation between the optical tracking and robot frame, ${}^{R}{\boldsymbol{R}_{OT}}$, and the rigid body relationship between the robot end-effector and the hemisphere, ${}^{EE}{\boldsymbol{T}_{HS}}$. The first stage ascertains ${}^{R}{\boldsymbol{R}_{OT}}$ and ${}^{EE}{\boldsymbol{R}_{HS}}$, and the second stage ascertains ${{}^{EE}\boldsymbol{t}}_{EE\to HS}$.


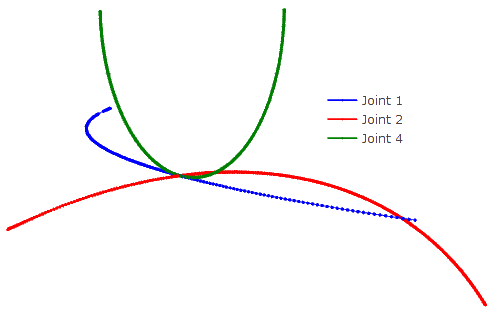


**X**

**Y**

**Z**

Fig. S1.1: Trajectories traced by the hemisphere rigid body as the robot rotates its 4^th^ (green), 2^nd^ (red), and 1^st^ (blue) joint. Vectors perpendicular to the plane fit of each trajectory define the robot's X, Y, and Z axis, respectively.

First, from the home position [1] the robot is instructed to sequentially rotate its 4^th^, 2^nd^, and 1^st^ joint by 90°, 70°, and 180° respectively. Meanwhile, the pose of the hemisphere, ${{}^{OT}\boldsymbol{T}}_{HS}$, is recorded using the optical tracking system. The vector perpendicular to a plane fit of the hemisphere position, ${}^{OT}{\boldsymbol{t}_{OT\to HS}}$, during sequential rotations corresponds to the robot’s X, Y, and Z axis, respectively (Fig. S1.1). This establishes the orientation of the robot frame within the optical tracking frame, ${}^{OT}{\boldsymbol{R}_{R}}$, which yields ${}^{R}{\boldsymbol{R}_{OT}}$:

$$\begin{aligned} {{}^{R}\boldsymbol{R}}_{OT}={{({}^{OT}\boldsymbol{R}}_{R})}^{T}\#\left( S1.1 \right) \end{aligned}$$

Because this is a measured, not computed, rotation matrix it is not guaranteed to be orthogonal. Orthogonality is enforced by projecting it to the nearest rotation matrix using the singular value decomposition [2]. Since in the home (0) position the rotation between the robot and end-effector frames is known (${}^{R}{\boldsymbol{R}_{EE}^{0}}$), this step also yields the end-effector orientation in the optical tracking frame while in the home (0) position, ${}^{OT}{\boldsymbol{R}_{EE}^{0}}$:

$$\begin{aligned} {{}^{OT}\boldsymbol{R}}_{EE}^{0}={{}^{OT}\boldsymbol{R}}_{R}\cdot{{}^{R}\boldsymbol{R}}_{EE}^{0}\#\left( S1.2 \right) \end{aligned}$$

The orientation of the hemisphere relative to the end-effector is subsequently computed as:

$$\begin{aligned} {{}^{EE}\boldsymbol{R}}_{HS}={{({}^{OT}\boldsymbol{R}}_{EE}^{0})}^{T}\cdot{{}^{OT}\boldsymbol{R}}_{HS}^{0}\#\left( S1.3 \right) \end{aligned}$$

Second, the robot is instructed to rotate, without translating, about its end-effector origin while in the home position. Again, the pose of the hemisphere, ${{}^{OT}\boldsymbol{T}}_{HS}$, is recorded using the optical tracking system. While the robot performs this motion, the origin of the hemisphere traces a partial sphere through space (Fig. S1.2). The centroid of a sphere fit to the generated point cloud establishes the inter-origin vector from the optical tracking frame to the end-effector frame in the home (0) position, ${{}^{OT}\boldsymbol{t}}_{OT\to EE}^{0}$. The inter-origin vector from the end-effector to the hemisphere frame can be computed as:


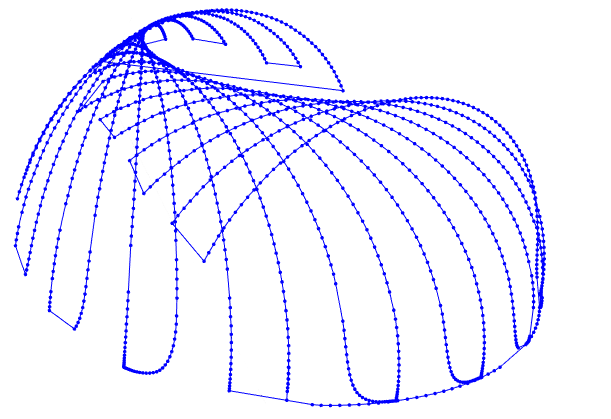


Fig. S1.2: Point cloud generated by the origin of the hemisphere rigid body as the robot rotates (without translating) around its end-effector.

$$\begin{aligned} {{}^{EE}\boldsymbol{t}}_{EE\to HS}={{({}^{OT}\boldsymbol{R}}_{EE}^{0})}^{T}\cdot\left( {{}^{OT}\boldsymbol{t}}_{OT\to HS}^{0}-{{}^{OT}\boldsymbol{t}}_{OT\to EE}^{0} \right)\#\left( S1.4 \right) \end{aligned}$$

Equations (S1.3) and (S1.4) compute the orientation and position of the hemisphere in the end-effector frame, respectively, thus determining the pose of the hemisphere with respect to the end-effector, ${}^{EE}{\boldsymbol{T}_{HS}}$.

1. **Procedure Results**

The robot reference frame identification procedure was performed at least once during any data collection session, which could include multiple trials. Even though the singular value decomposition was used to project the measured robot axes onto the nearest orthogonal matrix, this step was performed for the sake of mathematical correctness: the measured robot axes were very nearly orthogonal. The highest measured difference from orthogonality occurred between the Y and Z axis (corresponding to Joint 2 and 1, respectively) with a value of 0.46°. The median difference from orthogonal for the X and Y, X and Z, and Y and Z axis pairs were -0.005°, -0.033°, and 0.004° (Fig. S1.3).


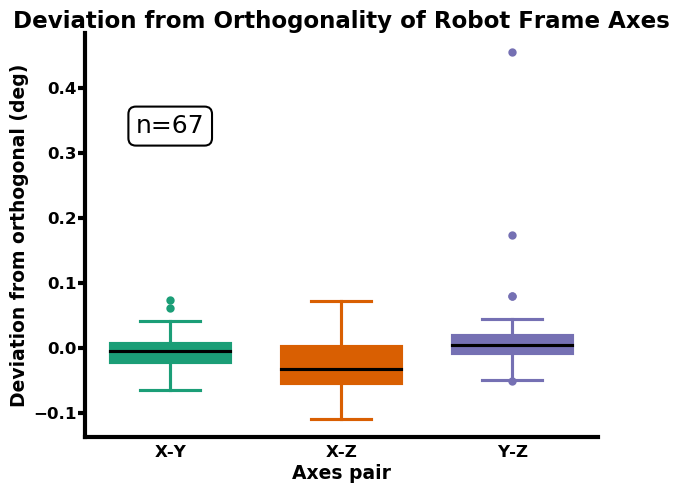


Fig. S1.3: Deviation from orthogonal of the robot's frame axes as determined from the robot reference frame identification procedure.

The rigid body relationship between the hemisphere and the robot end-effector, ${}^{EE}{\boldsymbol{T}_{HS}}$, is constant. The precision of the robot reference frame identification procedure at ascertaining this relationship can therefore be quantified. The predicted hemisphere positions relative to the robot-effector frame were normalized with respect to their mean, and the magnitude of the resulting positions was computed utilizing the Euclidean norm. The highest magnitude difference from the mean predicted position was 0.75 mm, with a median of 0.27 mm (Fig. S1.4A). Likewise, the predicted hemisphere orientations were normalized with respect to their mean, (see Markley et al. [3] for averaging orientations) and the magnitude of the resulting orientations was computed utilizing the geodesic distance of normed quaternions [4]. The highest magnitude difference from the mean was 0.65°, with a median of 0.28° (Fig. S1.4B).


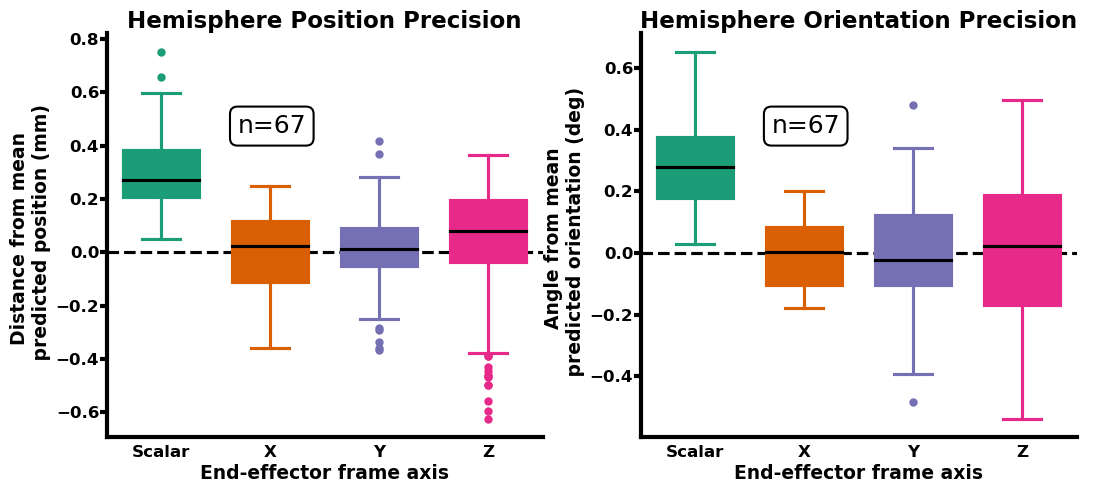


**A**

**B**

Fig. S1.4: Hemisphere position (**A**) and orientation (**B**) precision measured relative to the end-effector reference frame. The X, Y, Z components of orientation were computed from the xy’z’’ Cardan angle decomposition of the hemisphere orientation relative to the end-effector frame.

1. **References**

1. Craig JJ. Introduction to robotics: mechanics and control, 3/E: Pearson Education India; 2009.

2. Eggert DW, Lorusso A, Fisher RB. Estimating 3-D rigid body transformations: a comparison of four major algorithms. Machine Vision and Applications. 1997;9(5):272-90. doi: 10.1007/s001380050048.

3. Markley FL, Cheng Y, Crassidis JL, Oshman Y. Averaging quaternions. Journal of Guidance, Control, and Dynamics. 2007;30(4):1193-7.

4. Huynh DQ. Metrics for 3D Rotations: Comparison and Analysis. Journal of Mathematical Imaging and Vision. 2009;35(2):155-64. doi: 10.1007/s10851-009-0161-2.
