# Supplementary material for: Replicating dynamic humerus motion using an industrial robot
Source: PLoS One. 2020 Nov 9;15(11):e0242005. doi: 10.1371/journal.pone.0242005 (PMC7652298; doi:10.1371/journal.pone.0242005)
Supplement: S2 Appendix — (DOCX) [file pone.0242005.s002.docx]

**S2 Appendix - Derivative-Free Optimization Algorithm**

1. **Preliminary Concepts**
   1. **Inverse Kinematics**

This section reviews preliminary concepts on creating a joint space trajectory from an operational space trajectory [1]. These concepts should be well-known to readers with a robotics background but could be unfamiliar to readers in the field of biomechanics.

Let

 denote the vector of the concatenated end-effector linear and angular velocity vectors:

|  |  | (S2.1) |
| --- | --- | --- |

Here,

 denotes the end-effector linear velocity and

 denotes the end-effector angular velocity. Likewise, let

 denote the vector of robot joint angles and

 denote the vector of robot joint velocities, where

 represents the number of degrees of freedom of the robotic manipulator. For the robotic manipulator used in this study,

.

Then the differential kinematics equation:

|  |  | (S2.2) |
| --- | --- | --- |

provides a linear mapping from the robot joint velocities to the end-effector velocity in terms of the geometric Jacobian,

 [1]. This relationship can be inverted to obtain joint velocities from the end-effector velocity:

|  |  | (S2.3) |
| --- | --- | --- |

The geometric Jacobian of the robotic manipulator in this study is a square matrix so it can be inverted when non-singular. Solutions for dealing with non-square Jacobian matrices exist but they are beyond the scope of this manuscript [1]. To deal with singularities the Jacobian pseudo-inverse (

) is utilized, which solves Equation (S2.3) in the least-squares sense [2]. Near a singularity, both the Jacobian inverse and pseudo-inverse lead to very high joint velocities. A variety of techniques (e.g. damped least-squares [2]) exist to address this issue but these are not necessary as part of the presented algorithm because high joint velocities are penalized via a cost function.

If the initial manipulator posture

 is known, joint angles can be computed by integrating their velocities over time [1]:

|  |  | (S2.4) |
| --- | --- | --- |

The integration above can be performed in discrete time by utilizing numerical techniques such as the Euler integration method (

) [1]:

|  |  | (S2.5) |
| --- | --- | --- |

Combining equations (S2.3) and (S2.5), but using the Jacobian pseudo-inverse (

) instead of the Jacobian inverse (

) leads to:

|  |  | (S2.6) |
| --- | --- | --- |

The Euler method can be derived from the forward finite difference formula, which is used to approximate

:

|  |  | (S2.7) |
| --- | --- | --- |

Combining equations (S2.6) and (S2.7) leads to a recursive formula for obtaining a joint space trajectory,

, from an end-effector trajectory,

, given an initial manipulator posture,

:

|  |  | (S2.8) |
| --- | --- | --- |

- 1. **Orientation Error**

Although it is evident how to compute

 for position, a method for computing

 for orientation must be established. Since Equation (S2.8) is the discretized version of Equation (S2.3) the orientation components of

 should equal

 as

.

Let

 be the rotation matrix trajectory representing the orientation of the end-effector (EE) with respect to the base of the robot (R). Let

 be the angular velocity vector of

 evaluated at time

. Likewise, let

 be the rotation vector [3] of

 evaluated at time

. Then

 should be computed as:

|  |  | (S2.9) |
| --- | --- | --- |

In general, the rotation vector velocity -

 - is not equal to the angular velocity vector, but they are related by the relationship in Equation (S2.10) [4].

|  |  | (S2.10) |
| --- | --- | --- |

Now let the first argument of

,

, and

 be

. Note that

. Hence Equation (S2.10) becomes:

|  |  | (S2.11) |
| --- | --- | --- |

The simplification of Equation (S2.11) is proved at the end of this Appendix in Lemma 1.

Equation (S2.12) computes

, again, letting

:

|  |  | (S2.12) |
| --- | --- | --- |

Hence, in the limit

 Equation (S2.12) computes

, justifying Equation (S2.9).

It's insightful to note that:

|  |  | (S2.13) |
| --- | --- | --- |

Equation (S2.13) demonstrates that

 represents the rotation of the end-effector from time

 to

 in the reference frame of the robot (R).

- 1. **Joint Space and Operational Space Trajectory Computations**

Equation (S2.8) in Section 1.1 proposes a recursive formula for obtaining a joint space trajectory from an end-effector trajectory, given an initial manipulator posture. In this section, detailed steps for implementing this recursive procedure are provided.

The motion capture trajectories in this study are comprised of the pose of the humerus (H) measured in the motion capture (MC) laboratory frame of reference, encompassed in a 4x4 homogeneous transformation matrix, at equally spaced intervals of time:

,

,...,

 - where

 denotes the number of timepoints. Since the rigid-body relationship between the end-effector (EE) and the humerus (H) attached to the robot is known,

, then the pose of the end-effector in the motion capture reference frame is:

|  |  | (S2.14) |
| --- | --- | --- |

Given an initial manipulator posture

, the forward kinematics function of the robot,

, is utilized to compute the initial pose of the end-effector [1]:

|  |  | (S2.15) |
| --- | --- | --- |

The pose of the end-effector at each subsequent timepoint can be computed from its rigid-body relationship with the initial end-effector pose:

|  |  | (S2.16) |
| --- | --- | --- |

The use of a superscripted 'D' denotes that this is the desired operational space trajectory for which we seek a suitable joint space trajectory. Several factors, such as geometric Jacobian singularities and drifting away from the solution due to linearization, can prevent finding a joint space trajectory that corresponds to the desired operational space trajectory [1]. The operational space trajectory that corresponds to the joint space trajectory will be called the *achieved operational space trajectory* and will be denoted with a superscripted 'A'. Note that for the initial timepoint the desired and achieved end-effector poses are necessarily equal:

 as defined in Equation (S2.15).

To build a joint-space trajectory given an initial manipulator posture

 and corresponding actual operational space trajectory these steps are implemented recursively:

1. Compute the Jacobian pseudo-inverse at the current manipulator posture:

.
2. Compute the next manipulator posture according to equations (S2.8) and (S2.9):

|  |  | (S2.17) |
| --- | --- | --- |

1. Compute the achieved end-effector pose using the robot's forward kinematics function:

|  |  | (S2.18) |
| --- | --- | --- |

Again, it is insightful to note that:

|  |  | (S2.19) |
| --- | --- | --- |

Equation (S2.19) demonstrates that

 represents the rotation from the achieved end-effector pose at time

 to the desired end-effector pose at time

 in the robot reference frame.

1. **Optimization Problem**

Steps 1-3, along with Equations (S2.14), (S2.15), and (S2.16), provide a recursive relationship for computing a joint-space trajectory

 and corresponding achieved operational space trajectory

 from an initial manipulator posture

 and the humerus trajectory in the motion capture reference frame

. The problem at hand then is to determine an optimal initial manipulator posture

 that preserves the kinetic properties of the humerus trajectory

 while minimizing the effort expended by the robot. Given that the forward kinematics function,

, is non-linear the problem naturally lends itself to utilizing a non-linear optimizer.

In the formulation of this study, the parameters of the optimization problem are the joint angles of the initial manipulator posture,

. Since there are only six parameters, and because computation of the analytical gradient as the optimization problem is formulated is exceedingly difficult, a gradient-free non-linear numerical optimization method was selected. Specifically, the Constrained Optimization by Linear Approximation (COBYLA) method [5] as implemented in the NLopt library [6] within the Pagmo2 library [7] was utilized. The following sections describe the bounds, (in)equality constraints, and the objective function of the optimization. In all sections the degrees of freedom of the robot is

, while the number of timepoints in the trajectory is

.

A starting guess is necessary to seed the optimizer. This could be an educated guess based on the trajectory being optimized, or simply the home position of the robot. In our experience, the non-linear optimizer is robust. It finds the same initial manipulator posture even when the seeds are a large distance away from each other in joint space, although global optimality is not guaranteed. The provided seed (initial manipulator posture

), is evaluated for its fitness in faithfully reproducing the motion capture trajectory and minimizing the effort expended by the robot. The results are utilized as feedback in selecting the next manipulator posture for evaluation and a new optimization iteration begins. The optimization process stops when a maximum number of iterations has been reached, or when the change in

 or in the objective function (described below) from one iteration to the next reaches a defined tolerance.

Note that in the following sections the rotation vector function,

, is overloaded to accept just a proper orthogonal matrix instead of needing to specify an entire trajectory and the timepoint within the trajectory. Although the prior notation elucidated the proper calculation of orientation error, practically this function only needs to accept one argument.

- 1. **Objective Function**

The objective function determines the fitness of the joint space trajectory in accomplishing an objective, namely, minimizing the robot's joint utilization (measured as a percentage of the joint velocity limits) for the entire trajectory. Specifically, the goal is to minimize Equation (S2.20) whose summand is the cost function that maps a joint velocity percentage to a cost (Fig. S2.1). Let

 and

 denote the robot joint velocity limits, then:

|  |  | (S2.20) |
| --- | --- | --- |


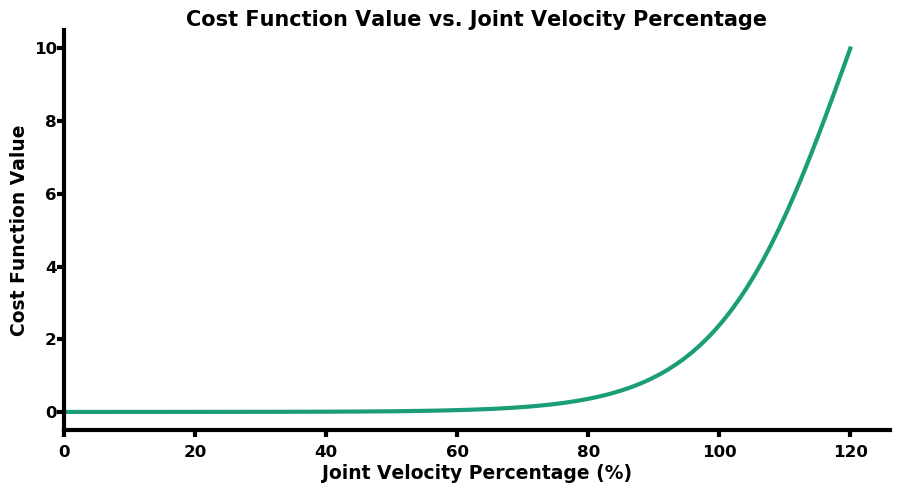


Fig. S2.1: Plot of cost function versus joint velocity percentage

- 1. **Bounds**

The robot joint angles of initial manipulator posture are the parameters of the optimization problem. Therefore, the lower and upper bounds for the algorithm are comprised of the robot joint limits. If the lower and upper joint limits of the robot are denoted by

 and

, respectively, then:

|  |  | (S2.21) |
| --- | --- | --- |

- 1. **Equality Constraints**

1. This equality constraint verifies that the desired and achieved operational space trajectories are congruent. It assures that there is no appreciable drift due to linearization and that solutions that pass through singularities are avoided.

|  |  | (S2.22) |
| --- | --- | --- |

1. This equality constraint assures that the implied rotation from the motion capture frame to the robot frame

 happens about the gravitational axis. By only allowing rotations (and any translations) about the gravitational axis, the kinetic properties of the trajectory are conserved. Letting the z-axis coincide with the gravitational axis with

:

|  |  | (S2.23) |
| --- | --- | --- |

- 1. **Inequality Constraints**

1. Since the bounds of the optimization problem only restrict the initial manipulator posture to within the robot joint limits, inequality constraints on the joint space trajectory are utilized to respect the robot joint limits for the entire trajectory:

|  |  | (S2.24) |
| --- | --- | --- |

1. This inequality constraint assures that the joint space trajectory respects the robot joint velocity limits:

|  |  | (S2.25) |
| --- | --- | --- |

1. The optimization algorithm can be instructed to stay within a rectangular prism in order to avoid obstacles in the laboratory. The boundaries of this rectangular prism are specified to the optimization algorithm via two of its corners and such that:

|  |  | (S2.26) |
| --- | --- | --- |

1. **Proofs**
   1. **Lemma 1**

Let and be proper orthogonal matrices, then:

|  |  | (S2.27) |
| --- | --- | --- |

*Proof:* For a trajectory , the angular velocity vector is the axial vector associated with the skew-symmetric angular velocity tensor [8]:

|  |  | (S2.28) |
| --- | --- | --- |

For a trajectory the angular velocity tensor is:

|  |  | (S2.29) |
| --- | --- | --- |

Since the angular velocity tensors for and are equal then their corresponding angular velocity axial vectors - and , respectively, must be equal.

1. **References**

1. Siciliano B, Sciavicco L, Villani L, Oriolo G. Robotics: modelling, planning and control: Springer Science & Business Media; 2010.

2. Buss SR. Introduction to inverse kinematics with jacobian transpose, pseudoinverse and damped least squares methods. IEEE Journal of Robotics and Automation. 2004;17(1-19):16.

3. Diebel J, editor Representing Attitude : Euler Angles , Unit Quaternions , and Rotation Vectors2006.

4. Peres A. Finite rotations and angular velocity. American Journal of Physics. 1980;48:70-1.

5. Powell MJD. A Direct Search Optimization Method That Models the Objective and Constraint Functions by Linear Interpolation. In: Gomez S, Hennart J-P, editors. Advances in Optimization and Numerical Analysis. Dordrecht: Springer Netherlands; 1994. p. 51-67.

6. Johnson SG. The NLopt nonlinear-optimization package. 2018.

7. Biscani F, Izzo D, Jakob W, Märtens M, Mereta A, Kaldemeyer C, et al. esa/pagmo2: pagmo 2.10. 2019.

8. Condurache D, Matcovschi M. Computation of angular velocity and acceleration tensors by direct measurements. Acta Mechanica. 2002;153(3):147-67. doi: 10.1007/bf01177449.
