# Supplementary material for: Replicating dynamic humerus motion using an industrial robot
Source: PLoS One. 2020 Nov 9;15(11):e0242005. doi: 10.1371/journal.pone.0242005 (PMC7652298; doi:10.1371/journal.pone.0242005)
Supplement: S3 Appendix — (DOCX) [file pone.0242005.s003.docx]

**S3 Appendix - Gradient Based Optimization Algorithm**

1. **Preliminary Concepts**

Please refer to Section 1 of Appendix S2 for a discussion on differential kinematics, angular velocity, and the rotation vector. As before, the function that extracts a rotation vector from a proper orthogonal matrix will be overloaded.

 extracts the rotation vector from the proper orthogonal matrix

, while

 extracts the rotation vector from the trajectory

 at time

. These amount to the same operation, but in the context of a discussion one may be more elucidating than the other.

1. **Optimization Problem**

The motion capture trajectories in this study are comprised of the pose of the humerus (H) measured in the motion capture (MC) laboratory frame of reference, encompassed in a 4x4 homogeneous transformation matrix, at equally spaced intervals of time:

,

,...,

 where

 denotes the number of timepoints. Since the rigid-body relationship between the end-effector and the humerus attached to the robot is known,

, then the pose of the end-effector in the motion capture reference frame is:

|  |  | (S3.1) |
| --- | --- | --- |

A transformation that will map the end-effector trajectory from the motion capture to the robot reference frame is desired. It can be shown that to preserve the kinetic properties of the trajectory this transformation must only admit rotations about the gravitational axis,

. Any reasonable translation

, which will anyhow be bounded by the size of the motion capture room and the robot working envelope, is also admissible.

|  |  | (S3.2) |
| --- | --- | --- |

The robot's forward kinematics function [1],

, permits reframing Equation (S3.2) in terms of the manipulator's joint angles vector,

:

|  |  | (S3.3) |
| --- | --- | --- |

Given that the forward kinematics function is non-linear, Equation (S3.3) can be formulated as a non-linear optimization problem. Let

 equal the number of timepoints in the motion capture trajectory and

 equal degrees of freedom of the robotic manipulator for all subsequent sections. To solve the non-linear optimization problem the Sparse Nonlinear Optimizer (SNOPT) software package [2], which utilizes a sparse sequential quadratic programming algorithm to minimize a nonlinear objective function, is utilized within the Pagmo2 library [3].

- 1. **Optimization Problem Formulation**

The parameters of the optimization problem are the robot joints angles at each timepoint, as well as

 and

. Equation (S3.4) represents the optimization parameters in vector form. Note that there are

 parameters.

|  |  | (S3.4) |
| --- | --- | --- |

From this vector a desired (D) and achieved (A) end-effector trajectory is extracted.

|  |  | (S3.5) |
| --- | --- | --- |
|  |  | (S3.6) |

- 1. **Objective Function**

The objective function determines the fitness of the joint space trajectory in accomplishing an objective, namely, minimizing the robot's joint utilization (measured as a percentage of the joint velocity limits) for the entire trajectory. From

, joint velocities are computed using the central difference formula:

|  |  | (S3.7) |
| --- | --- | --- |

Joint velocities for each time point are normalized based on the maximum joint velocity, and a logistic cost function is utilized to associate a cost to each normalized joint velocity (Fig. S3.1).

|  |  | (S3.8) |
| --- | --- | --- |


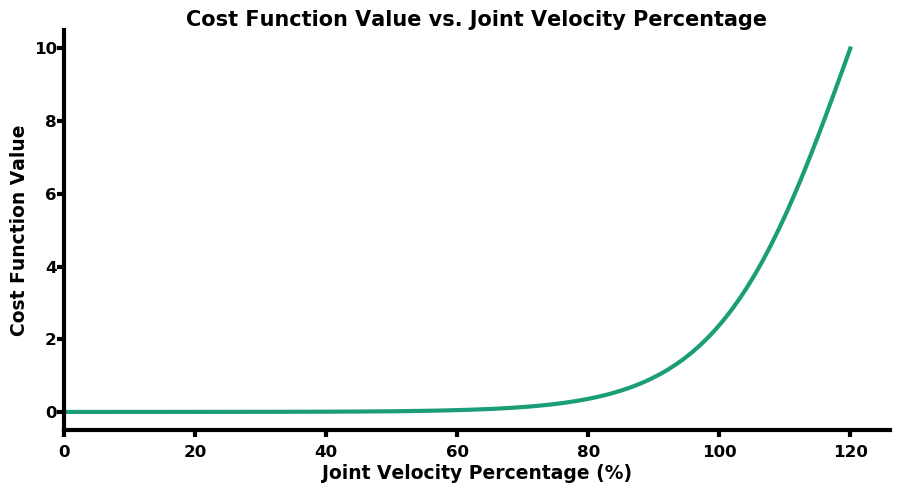


Fig. S3.1: Plot of logistic cost function versus joint velocity percentage

Specifically, the goal is to minimize the objective function presented in Equation (S3.9).

|  |  | (S3.9) |
| --- | --- | --- |

- 1. **Bounds**

If the lower and upper joint limits of the robot are denoted by

 and

, respectively, then:

|  |  | (S3.10) |
| --- | --- | --- |

Note that

 is specified in radians while

 is specified in meters. These parameters are configurable.

- 1. **Equality Constraints**

Each end-effector pose

 is comprised of a translation vector,

, and a rotation matrix,

. The equality constraint assures that the desired (D) and achieved (A) trajectories are congruent.

|  |  | (S3.11) |
| --- | --- | --- |

- 1. **Inequality Constraints**

The inequality constraint assures that the joint space trajectory respects the robot joint velocity limits:

|  |  | (S3.12) |
| --- | --- | --- |

1. **Optimization Problem Gradients**

The large dimensionality of the optimization parameters vector (

) necessitates the use of a gradient. The trajectories in this study can contain anywhere between 200-500 timepoints equating to an optimization parameter vector of 1200 to 3000 dimensions. In this section the gradient associated with the objective function and (in)equality constraints is derived.

- 1. **Objective Function Gradient**

The objective function does not depend on

 or

, hence:

|  |  | (S3.13) |
| --- | --- | --- |
|  |  | (S3.14) |

The derivative of a logistic function,

, is:

|  |  | (S3.15) |
| --- | --- | --- |

According to Equation (S3.7), every

 affects both

 and

, where the indices

 and

 are dictated by

. Equation (S3.16) computes

 for generic indices

 and

. To ease notation let

 be denoted by

.

|  |  | (S3.16) |
| --- | --- | --- |

Then, when

:

|  |  | (S3.17) |
| --- | --- | --- |

When

:

|  |  | (S3.18) |
| --- | --- | --- |

When

:

|  |  | (S3.19) |
| --- | --- | --- |

- 1. **Equality Gradient**

The equality gradient will be computed for separately for the position

 and orientation

 components with respect to

,

, and

. First, it's useful to compute the derivative of the dot product of a vector that depends on a tensor of any order (this includes scalars, vectors, etc.),

, with itself. We switch to indicial notation briefly for ease of notation.

|  |  | (S3.20) |
| --- | --- | --- |

Also, it is useful to write Equation (S3.5) in expanded matrix form:

|  |  | (S3.21) |
| --- | --- | --- |



 represents a rotation about the z-axis by

. In this study the robot z-axis and the motion capture z-axis are coincident with the gravitational axis.

|  |  | (S3.22) |
| --- | --- | --- |

Equation (S3.21) gives a formula for

:

|  |  | (S3.23) |
| --- | --- | --- |

Likewise, for orientation:

|  |  | (S3.24) |
| --- | --- | --- |

Equation (S3.25) computes the derivative of Equation (S3.22) with respect to

:

|  |  | (S3.25) |
| --- | --- | --- |

- - 1. **Position Equality Gradient**

Equation (S3.26) computes the position equality gradient with respect to

.

|  |  | (S3.26) |
| --- | --- | --- |

Equation (S3.27) computes the position equality gradient with respect to

.

|  |  | (S3.27) |
| --- | --- | --- |

And finally, Equation (S3.28) computes the position equality gradient with respect to

.

|  |  | (S3.28) |
| --- | --- | --- |

- - 1. **Orientation Equality Gradient**

Note that since the derivative of the orientation portion of Equation (S3.11) is taken with respect to

,

becomes a trajectory that's dependent on

. To ease notation, let

 and write

. Also recall the following equalities from Appendix 2.

|  |  | (S3.29) |
| --- | --- | --- |
|  |  | (S3.30) |

The orientation portion of Equation (S3.11) is not dependent on

 therefore:

|  |  | (S3.31) |
| --- | --- | --- |

Equation (S3.32) computes the orientation equality gradient with respect to

, utilizing Equations (S3.29) and (S3.30) in its simplification.

|  |  | (S3.32) |
| --- | --- | --- |

Equations (S3.33) and (S3.34) provide support in computing the orientation equality gradient with respect to

. Equation (S3.33) utilizes Equation (S3.29), as well as Lemma 1, Lemma 2, and Lemma 3 (see end of Appendix) in its simplification.

|  |  | (S3.33) |
| --- | --- | --- |

Since Equation (S3.33) is valid for all then:

|  |  | (S3.34) |
| --- | --- | --- |

Hence the gradient of the orientation equality with respect to is:

|  |  | (S3.35) |
| --- | --- | --- |

- 1. **Inequality Gradient**

Just as with the objective function, the joint velocity inequality does not depend on or , hence:

|  |  | (S3.36) |
| --- | --- | --- |
|  |  | (S3.37) |

Equations (S3.38), (S3.39), and (S3.40) compute the inequality derivative of joint at time with respect to joint at time , when , , and , respectively.

|  |  | (S3.38) |
| --- | --- | --- |
|  |  | (S3.39) |
|  |  | (S3.40) |

1. **Proofs**
   1. **Lemma 1**

Let and be proper orthogonal matrices, then:

|  |  | (S3.41) |
| --- | --- | --- |

*Proof*: For a trajectory , the angular velocity vector is the axial vector associated with the skew-symmetric angular velocity tensor [4]:

|  |  | (S3.42) |
| --- | --- | --- |

For a trajectory the angular velocity tensor is:

|  |  | (S3.43) |
| --- | --- | --- |

One could utilize the rules for extracting an axial vector from a skew-symmetric tensor to prove Equation (S3.41). However, it is just as easy to note that by tensor transformation laws [5], if transforms according to Equation (S3.43) then must transform according to Equation (S3.41).

- 1. **Lemma 2**

Let be a proper orthogonal matrix, then:

|  |  | (S3.44) |
| --- | --- | --- |

*Proof*: For a trajectory the angular velocity tensor is:

|  |  | (S3.45) |
| --- | --- | --- |

The first simplification steps of Equation (S3.45) relies on the fact that is orthogonal, so its inverse equals its transpose, and that for any invertible matrix :

|  |  | (S3.46) |
| --- | --- | --- |

One could utilize the rules for extracting an axial vector from a skew-symmetric tensor to prove Equation (S3.44). However, it is just as easy to note that by tensor transformation laws [5], if transforms according to Equation (S3.45) then must transform according to Equation (S3.44).

- 1. **Lemma 3**

Let be a proper orthogonal matrix, then:

|  |  | (S3.47) |
| --- | --- | --- |

Furthermore:

|  |  | (S3.48) |
| --- | --- | --- |

*Proof*: points in the direction of the unique axis that is fixed by both and proving Equation (S3.47) [6]. Equation (S3.48) follows directly from Equation (S3.47) by basic linear algebra.

1. **References**

1. Siciliano B, Sciavicco L, Villani L, Oriolo G. Robotics: modelling, planning and control: Springer Science & Business Media; 2010.

2. Gill PE, Murray W, Saunders MA. SNOPT: An SQP algorithm for large-scale constrained optimization. SIAM review. 2005;47(1):99-131.

3. Biscani F, Izzo D, Jakob W, Märtens M, Mereta A, Kaldemeyer C, et al. esa/pagmo2: pagmo 2.10. 2019.

4. Condurache D, Matcovschi M. Computation of angular velocity and acceleration tensors by direct measurements. Acta Mechanica. 2002;153(3):147-67. doi: 10.1007/bf01177449.

5. Spencer AJM. Continuum mechanics: Courier Corporation; 2004.

6. Diebel J, editor Representing Attitude : Euler Angles , Unit Quaternions , and Rotation Vectors2006.
