# Supplementary material for: Replicating dynamic humerus motion using an industrial robot
Source: PLoS One. 2020 Nov 9;15(11):e0242005. doi: 10.1371/journal.pone.0242005 (PMC7652298; doi:10.1371/journal.pone.0242005)
Supplement: S4 Appendix — (DOCX) [file pone.0242005.s004.docx]

**S4 Appendix -**

**Programming Optimized Humeral Trajectories on M20ia Robot**

In FANUC robotics, the CNT tag is the smoothness parameter that allows control over how closely the robot approaches a programmed point versus how much it decelerates [1]. A representative trial from each activity was programmed onto the robot using 11 different CNT tags, and the resulting motion was analyzed for position, orientation, linear/angular velocity and linear/angular acceleration mean absolute error (MAE). For all activities, and both linear and angular measures, CNT tags of 91,92,93,and 94 yielded similar results (Fig. S4.1). A CNT tag of 92 was selected for all motions as it provided the best trade off between accuracy and speed.


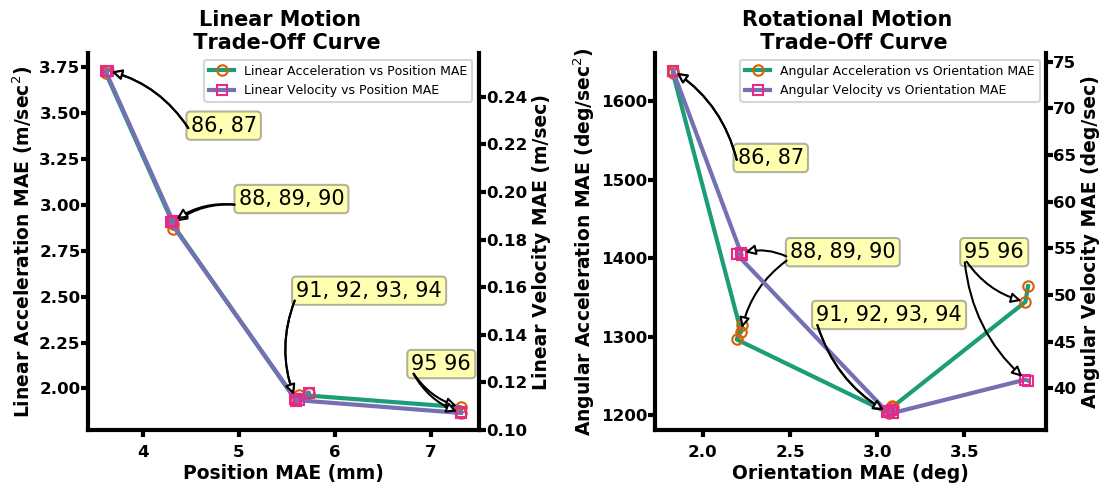


Fig. S4.1: Trade-off curves for linear and rotational motion for a representative jumping jack trial. The yellow highlighted labels point to the CNT tags examined.

The maximum velocity between two timepoints was determined empirically. First, the robot was programmed using the average velocity between two timepoints as calculated from the motion capture dataset, and the resulting motion was tracked using the optical tracking system. The programmed velocity was then iteratively scaled by the robot operator until corresponding points between the motion capture trajectory and the tracked robot trajectory, determined via dynamic time warping [2], were aligned in time.

**References**

1. McMahon I. Improving ROS-Industrial motion on an Industrial Robot (Implementation Notes) 2016 [cited 2020 February 10]. Available from: <http://wiki.ros.org/Industrial/Tutorials/Improving_Motion_on_an_Industrial_Robot>.

2. Aristidou A, Cohen‐Or D, Hodgins JK, Shamir A, editors. Self‐similarity analysis for motion capture cleaning. Computer Graphics Forum; 2018: Wiley Online Library.
