# Supplementary material for: Replicating dynamic humerus motion using an industrial robot
Source: PLoS One. 2020 Nov 9;15(11):e0242005. doi: 10.1371/journal.pone.0242005 (PMC7652298; doi:10.1371/journal.pone.0242005)
Supplement: S5 Appendix — (DOCX) [file pone.0242005.s005.docx]

**S5 Appendix - Non-Uniform Subsampling Algorithm**

1. **Subsampling Algorithm Description**

A heuristic non-uniform subsampling algorithm that removes relatively fewer points in regions of higher curvature was developed to preserve the geometry of the trajectory. A heuristic measure of curvature was utilized because it was desirable to avoid directly computing curvature. Its computation depends on two successive differentiations and is highly sensitive to noise present in the datase. Furthermore, it was desirable to consider orientations when subsampling the trajectory and to the authors’ knowledge a method for computing curvature for orientations does not exist.

The non-uniform subsampling algorithm is an iterative procedure that removes interior timepoints (any but the first and last) from a trajectory until the desired subsampling rate is reached. A timepoint herein refers to a sample from a discretized pose trajectory, therefore it encompasses both the position and orientation of a rigid body. Assume that adjacent discretized timepoints are connected via a linear path, which is a good assumption in a trajectory that has been discretized at a high frequency, and the simplest connectivity assumption. For linear motion, a linear path is intuitive. The analog for rotational motion is a rotation about a fixed axis. If a timepoint is deleted, then its two neighbors will be newly connected via a linear path. A natural measure for the error that is introduced by deleting a timepoint is the distance between the deleted timepoint and its orthogonal projection onto the linear path that now connects its two neighbors (Fig. S5.1). For linear motion the notion of Euclidean distance and the orthogonal projection computed via the dot product are sufficient in computing this error.

However, a metric and a method for computing orthogonal projections for orientations must be selected. Orthogonal projections for rotational motion can be computed by utilizing the swing-twist decomposition [1]. Let P1, P2, and P3 be three temporally sequential timepoints in a trajectory. Then the swing portion represents the rotation from P1 to P2 projected onto the axis of the rotation from P1 to P3. The twist portion represents the rotation from this projection to P2. The swing-twist decomposition guarantees that orthogonality of the swing and twist axes of rotation [1]. This decomposition then is analogous to the one utilized for linear motion. The geodesic distance of normed quaternions, which is the absolute value of the angle of rotation from one orientation to another via a fixed axis, was selected as a distance metric for rotational motion [2].


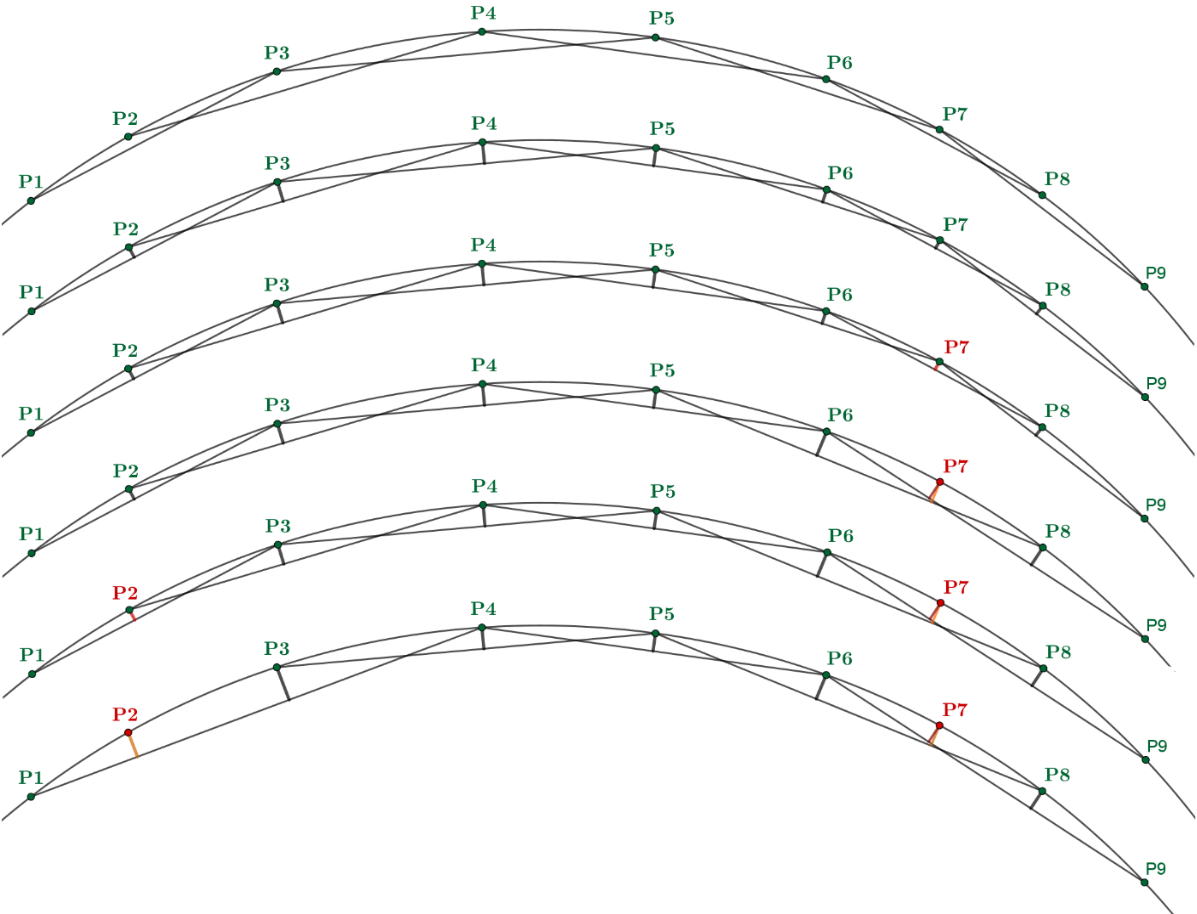


**1**

**2**

**3**

**4**

**5**

**6**

Fig. S5.1: Schematic of the non-uniform subsampling algorithm demonstrating how points are iteratively removed from a trajectory.

The steps of the heuristic non-uniform subsampling algorithm are:

1. Given a trajectory, the error associated with deleting any interior timepoint from the trajectory is computed.
   1. It is assumed that any trajectory timepoint and its 2^nd^ neighbor are connected via a linear path (Fig. S5.1, Step 1).
   2. The linear and rotational error is computed by determining the orthogonal distance between an interior timepoint and the linear path connecting its two neighbors (Fig. S5.1, Step 2).
   3. The linear and rotational errors associated with all the interior timepoints are ranked separately with a lower rank attributed to a lower error.
   4. The combined rank of a timepoint is computed as the maximum rank of its linear and rotational error.
2. The interior timepoint with the lowest combined error rank is deleted from the trajectory (Fig. S5.1, Step 3). It is now considered a deleted timepoint, while the remaining timepoints are considered active timepoints.

Steps 1 and 2 are repeated until the desired subsampling rate is achieved with the following new considerations:

1. A deleted timepoint is removed from consideration as a 2^nd^ neighbor. For example, the 2^nd^ neighbor of P6 is no longer P8 but P9 (Fig. S5.1, Step 4). Likewise, the 2^nd^ neighbor of P5 is now P8, not P7.
2. A linear path connecting 2^nd^ neighbors may now span multiple trajectory timepoints (one active timepoint and zero or more deleted timepoints). The error associated with removing the active timepoint is the maximum of the orthogonal distance of all the timepoints spanned by the linear path. For example, the linear path connecting P5 and P8 now spans the active timepoint P6 and the deleted timepoint P7. The error associated with deleting P6 is the maximum of the orthogonal distances of P6 and P7 to the linear path connecting P5 and P8.

This heuristic algorithm is based on two insights:

1. In a discretized trajectory represented by 3 timepoints, the perpendicular distance between timepoint 2 and the linear path connecting timepoints 1 and 3 is proportional to the curvature of the trajectory.
2. The perpendicular distance grows in proportion to the subsampling of the curve. For example, if P7 were not removed in Step 3 (Fig. S5.1), then P8 would be the next candidate for removal. However, since P7 was removed the perpendicular distance of P8 increases and P2 becomes the next candidate for removal.
3. **Subsampling Algorithm Results**

The trajectory maximum error and mean absolute error (MAE) was significantly different (Wilcoxon signed-rank test) between the non-uniform subsampling algorithm and uniform subsampling except for jug lifts (Fig. S5.2). Specifically, no significant difference was found for the maximum error of the position of the humerus and the MAE of the orientation of the humerus between uniform and non-uniform subsampling for jug lifts. This is likely due to the slower linear and angular velocity of jug lifts compared to all other activities. Since timepoints are more closely clustered together in a jug lift trajectory, it’s more likely that different subsampling strategies yield similar results. The non-uniform subsampling algorithm outperformed uniform subsampling in all measures except for the position MAE of jumping jacks and jug lifts, and the orientation MAE of jumping jacks (Fig. S5.2).This behavior is expected since the main goal of the non-uniform optimization algorithm is to closely mimic the curvature of the original trajectory. In doing so, the maximum error due to subsampling will be reduced but potentially accompanied with an increase in the MAE. Examining the position curvature of individual trajectories confirms that the non-uniform subsampling algorithm more closely matches the position curvature of the original trajectory (Fig. S5.3).


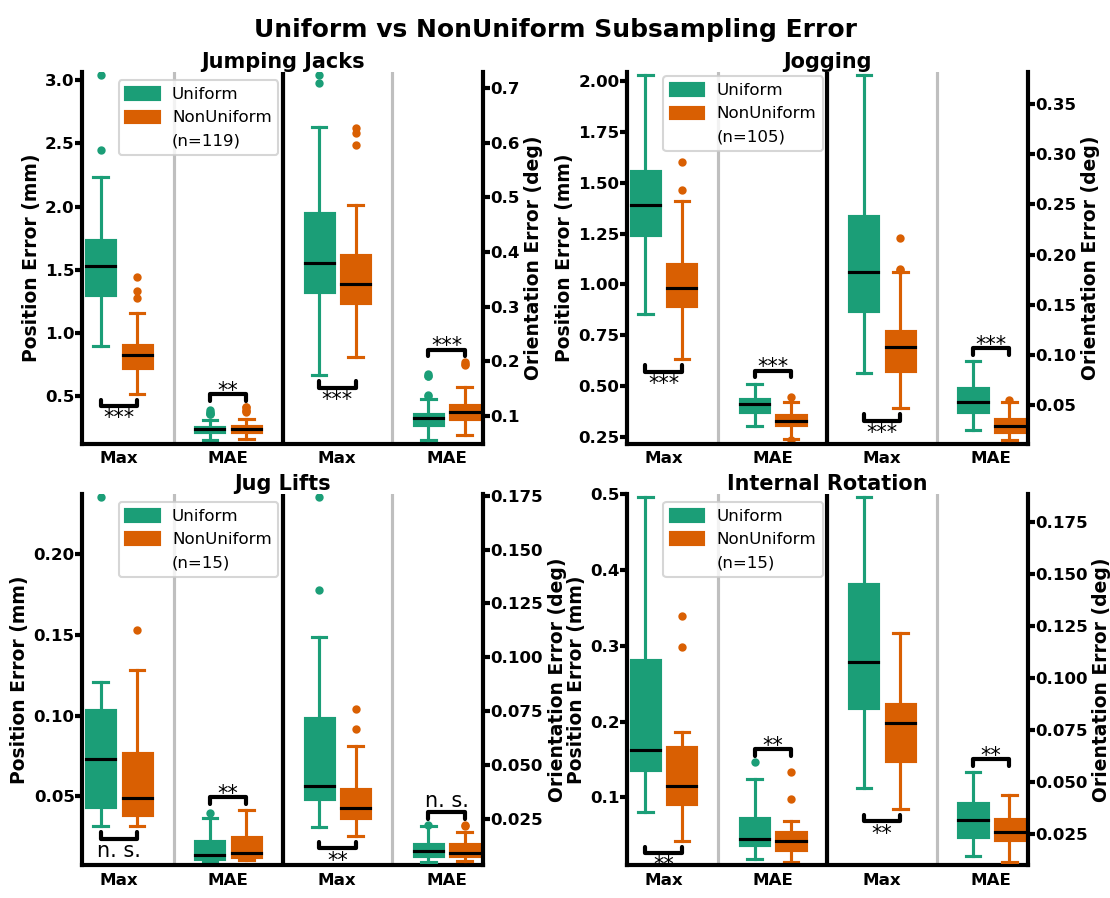


Fig. S5.2: Comparison of maximum error and MAE resulting from uniform versus non-uniform subsampling for all activities. N.s. stands for not significant; * indicates p≤0.05; ** p≤0.005; and *** p≤0.0005.


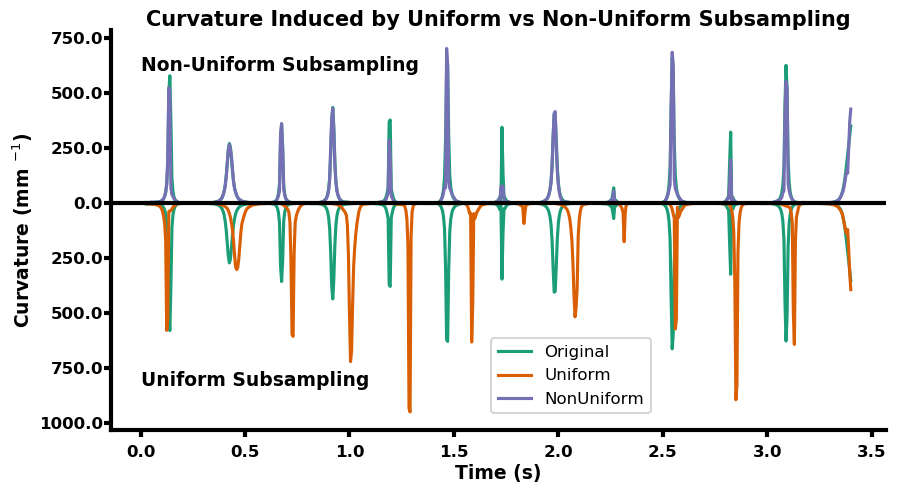


Fig. S5.3: Comparison of position curvature induced by uniform vs non-uniform subsampling for a representative jumping jack. The non-uniform subsampling trajectory curvature is shown at the top while the uniform subsampling trajectory curvature is shown at the bottom of the graph. The original trajectory curvature is mirrored across the x-axis for easy comparison against both subsampling methods.

1. **References**

1. Huyghe B. Design and implementation of a mobile sensor system for human posture tracking: Ghent University; 2011.

2. Huynh DQ. Metrics for 3D Rotations: Comparison and Analysis. Journal of Mathematical Imaging and Vision. 2009;35(2):155-64. doi: 10.1007/s10851-009-0161-2.
