# Supplementary material for: Replicating dynamic humerus motion using an industrial robot
Source: PLoS One. 2020 Nov 9;15(11):e0242005. doi: 10.1371/journal.pone.0242005 (PMC7652298; doi:10.1371/journal.pone.0242005)
Supplement: S6 Appendix — (DOCX) [file pone.0242005.s006.docx]

**S6 Appendix – Representative Trend Plots for All Activities**


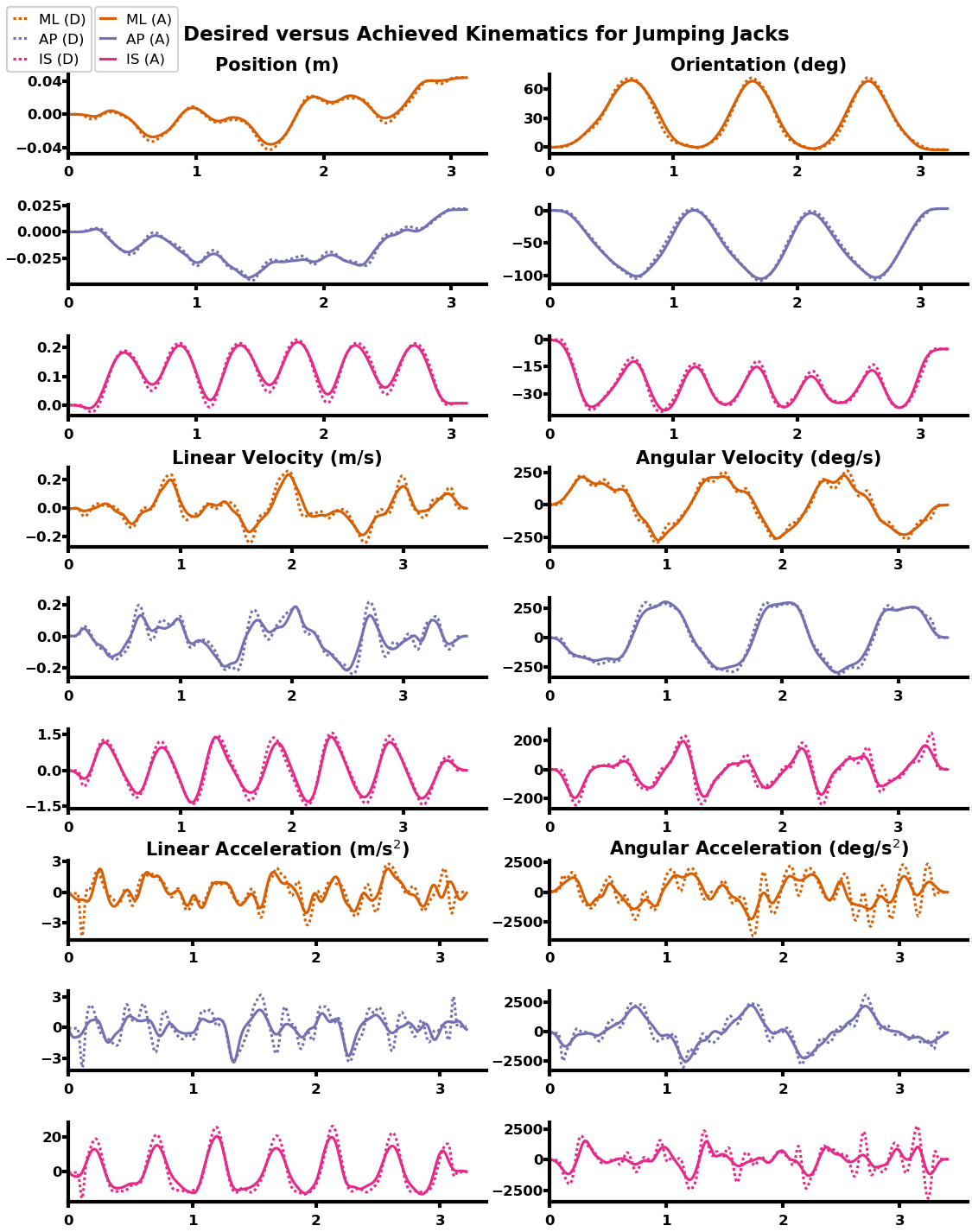


Fig. S6.1: Desired (D) versus achieved (A) kinematics for jumping jacks. For each kinematic variable desired versus achieved trajectories for the mediolateral (ML), anterior/posterior (AP), and inferior/superior (IS) are presented.


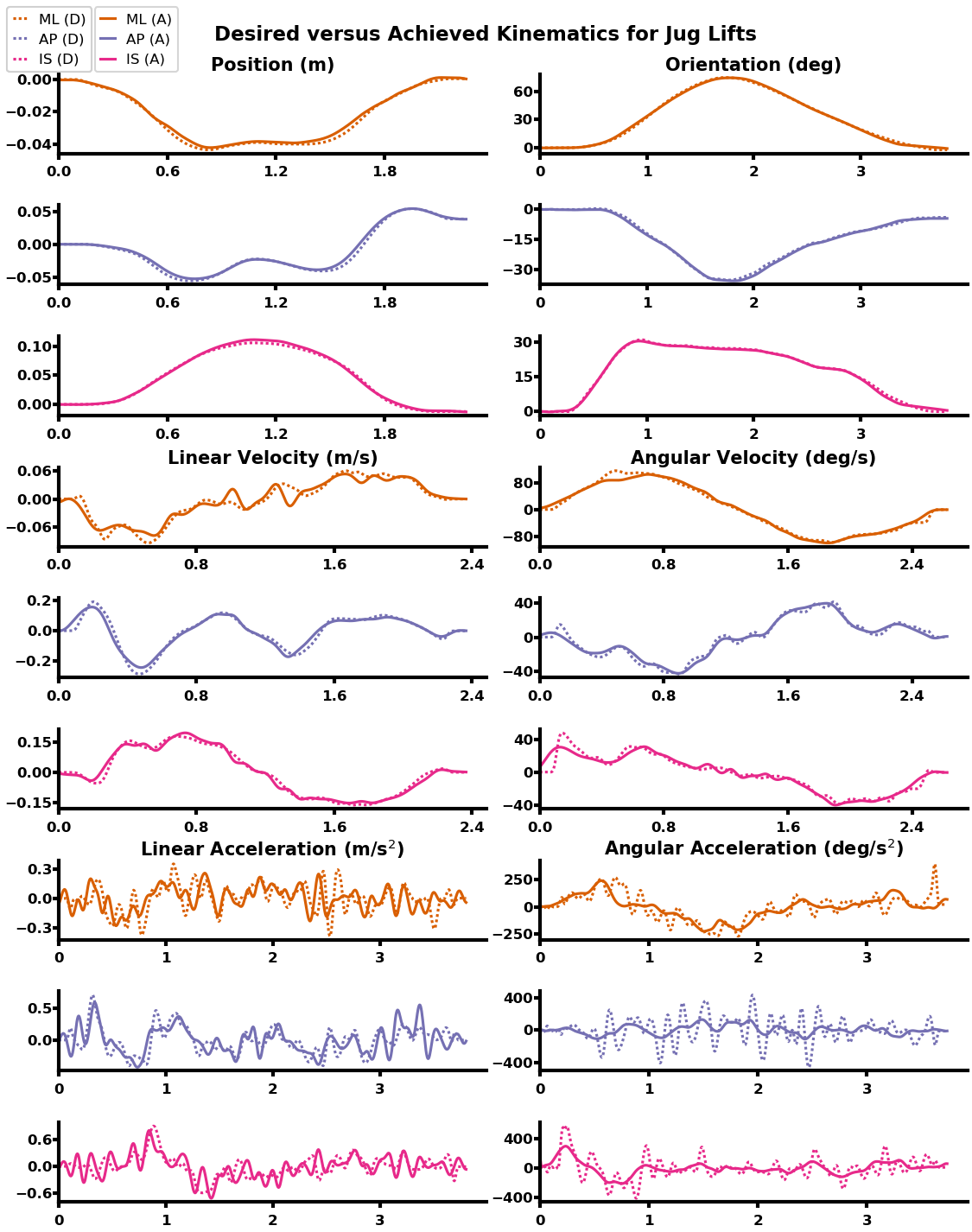


Fig. S6.2: Desired (D) versus achieved (A) kinematics for jug lifts. For each kinematic variable desired versus achieved trajectories for the mediolateral (ML), anterior/posterior (AP), and inferior/superior (IS) are presented.


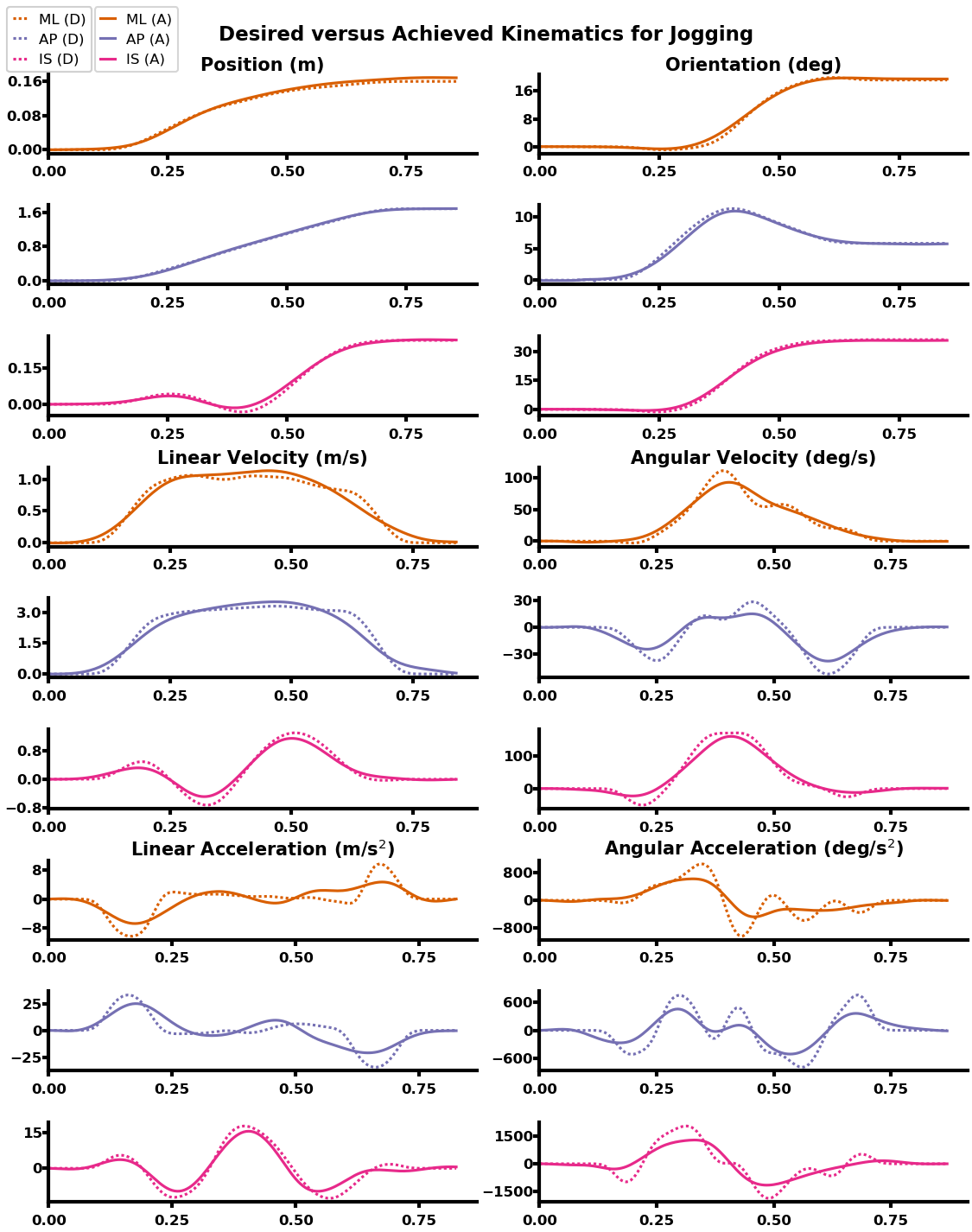


Fig. S6.3: Desired (D) versus achieved (A) kinematics for jogging. For each kinematic variable desired versus achieved trajectories for the mediolateral (ML), anterior/posterior (AP), and inferior/superior (IS) are presented.


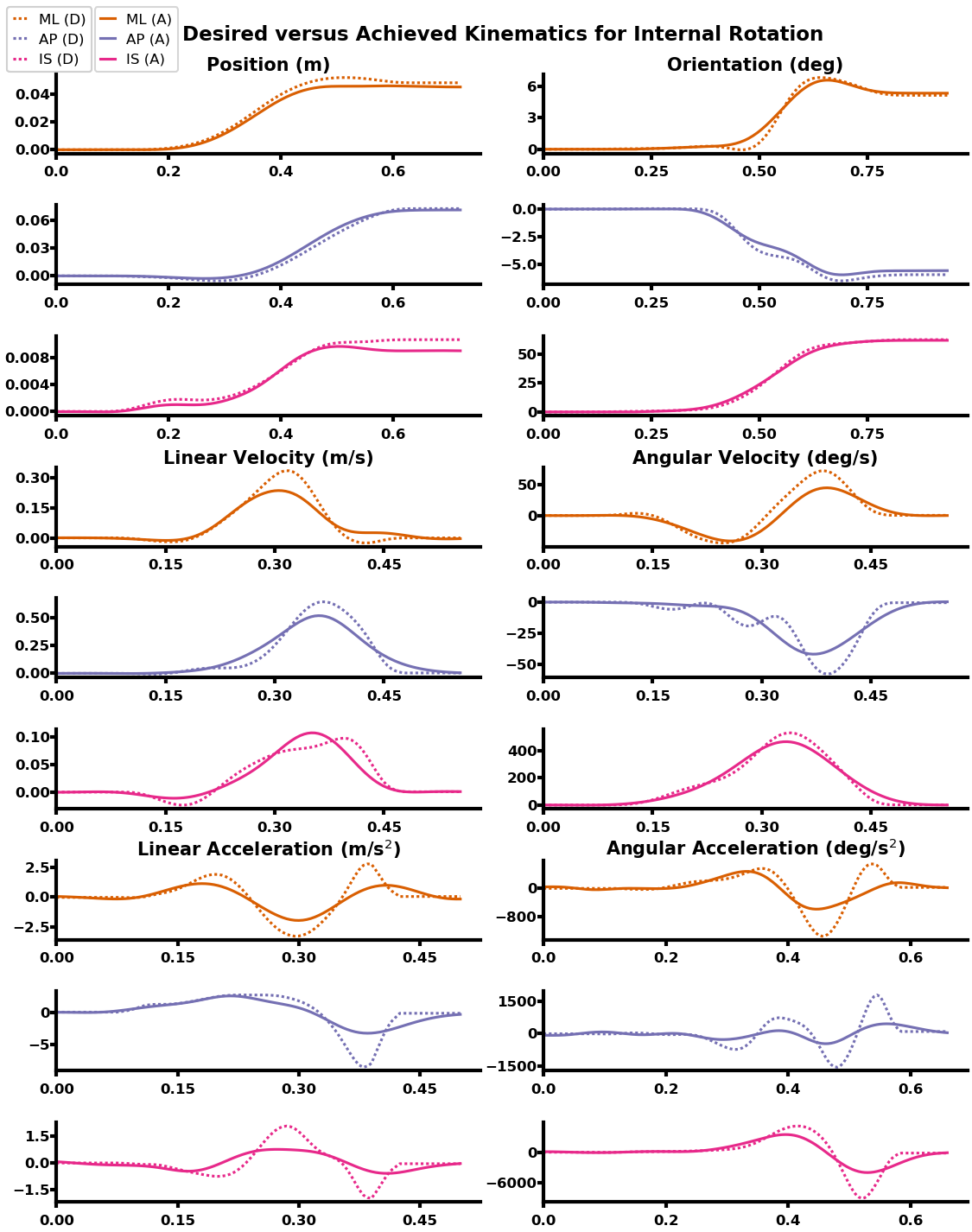


Fig. S6.4: Desired (D) versus achieved (A) kinematics for internal rotation. For each kinematic variable desired versus achieved trajectories for the mediolateral (ML), anterior/posterior (AP), and inferior/superior (IS) are presented.
